# Mosaic loss of Y chromosome is associated with aging and epithelial injury in chronic kidney disease

**DOI:** 10.1186/s13059-024-03173-2

**Published:** 2024-01-29

**Authors:** Parker C. Wilson, Amit Verma, Yasuhiro Yoshimura, Yoshiharu Muto, Haikuo Li, Nicole P. Malvin, Eryn E. Dixon, Benjamin D. Humphreys

**Affiliations:** 1 Division of Diagnostic Innovation, Department of Pathology and Laboratory Medicine, University of Pennsylvania, Philadelphia, PA USA; 2 Division of Nephrology, Department of Medicine, Washington University in St. Louis, St. Louis, MO USA; 3 Department of Developmental Biology, Washington University in St. Louis, St. Louis, MO USA

## Abstract

**Background:**

Mosaic loss of Y chromosome (LOY) is the most common chromosomal alteration in aging men. Here, we use single-cell RNA and ATAC sequencing to show that LOY is present in the kidney and increases with age and chronic kidney disease.

**Results:**

The likelihood of a cell having LOY varies depending on its location in the nephron. Cortical epithelial cell types have a greater proportion of LOY than medullary or glomerular cell types, which may reflect their proliferative history. Proximal tubule cells are the most abundant cell type in the cortex and are susceptible to hypoxic injury. A subset of these cells acquires a pro-inflammatory transcription and chromatin accessibility profile associated with expression of *HAVCR1*, *VCAM1*, and *PROM1*. These injured epithelial cells have the greatest proportion of LOY and their presence predicts future kidney function decline. Moreover, proximal tubule cells with LOY are more likely to harbor additional large chromosomal gains and express pro-survival pathways. Spatial transcriptomics localizes injured proximal tubule cells to a pro-fibrotic microenvironment where they adopt a secretory phenotype and likely communicate with infiltrating immune cells.

**Conclusions:**

We hypothesize that LOY is an indicator of increased DNA damage and potential marker of cellular senescence that can be applied to single-cell datasets in other tissues.

**Supplementary Information:**

The online version contains supplementary material available at 10.1186/s13059-024-03173-2.

## Background

Somatic mosaicism results from the accumulation of DNA damage over time, leading to a unique genotype in every cell [1]. Mutations can be as small as a single base pair or as large as an entire chromosome—affecting hundreds or even thousands of genes. The vast majority of mutations do not cause cancer but may affect tissue biology in other ways [2]. Mosaic chromosomal alterations (mCA) are a type of somatic mosaicism characterized by large chromosomal gains or losses. They have been associated with aging, heart disease, kidney disease, infection, cancer, and increased mortality [3]. Mosaic loss of Y chromosome (LOY) is the most common mCA in aging men, and the proportion of men with LOY in peripheral blood increases to more than 70% in the elderly population [4]. Although LOY was described more than 50 years ago in the blood [5] and bone marrow [6], more recently, it has also been detected in the brain [7, 8], buccal mucosa [9], bladder [10], and kidney [11, 12]. Conventional cytogenetics was the earliest method used to detect LOY, but with single-cell sequencing, it is now possible to measure mCA in hundreds of thousands of cells [13, 14]. Newer methods estimate single-cell karyotypes from sparse datasets and have mostly been used to study cancer [15]. Here, we describe an approach to detect LOY and other mCA by single-cell sequencing in human kidney cortex from donors with and without chronic kidney disease (CKD). These methods employ single nucleus multiome (10X Genomics, Single Cell Multiome), single nucleus ATAC (snATAC-seq), single-cell RNA sequencing (scRNA-seq), or digital PCR to estimate DNA damage burden and quantify its effects on cell-specific gene expression and chromatin accessibility.

CKD is an ideal model for studying mCA because the kidney is composed of more than twenty different cell types with varying susceptibility to injury and capacity for self-renewal [16]. The proximal tubule is the most abundant cell type in the kidney cortex and is highly susceptible to hypoxic injury, which leads to de-differentiation and cell division to repopulate the epithelium [17]. Cells are more vulnerable to DNA damage during cell division, and this process may lead to increased DNA damage during subsequent rounds of injury and repair—accompanied by a pro-inflammatory transcriptional and chromatin accessibility profile that predicts future kidney function decline [18, 19]. Along the same lines, cell cycle arrest in mouse models of acute kidney injury helps to mitigate DNA damage and prevent proximal tubule apoptosis [20]. Here, we show that epithelial injury, cell location, genomic instability, and age are all risk factors for LOY in the kidney. In addition, proximal tubule cells with LOY express pro-survival pathways that may help them to escape apoptosis. We hypothesize that LOY and somatic mosaicism is associated with cellular senescence and the establishment of a pro-fibrotic microenvironment that drives CKD progression.

## Results

### LOY detection by simultaneous single nucleus RNA and ATAC sequencing

**Fig. 1:**
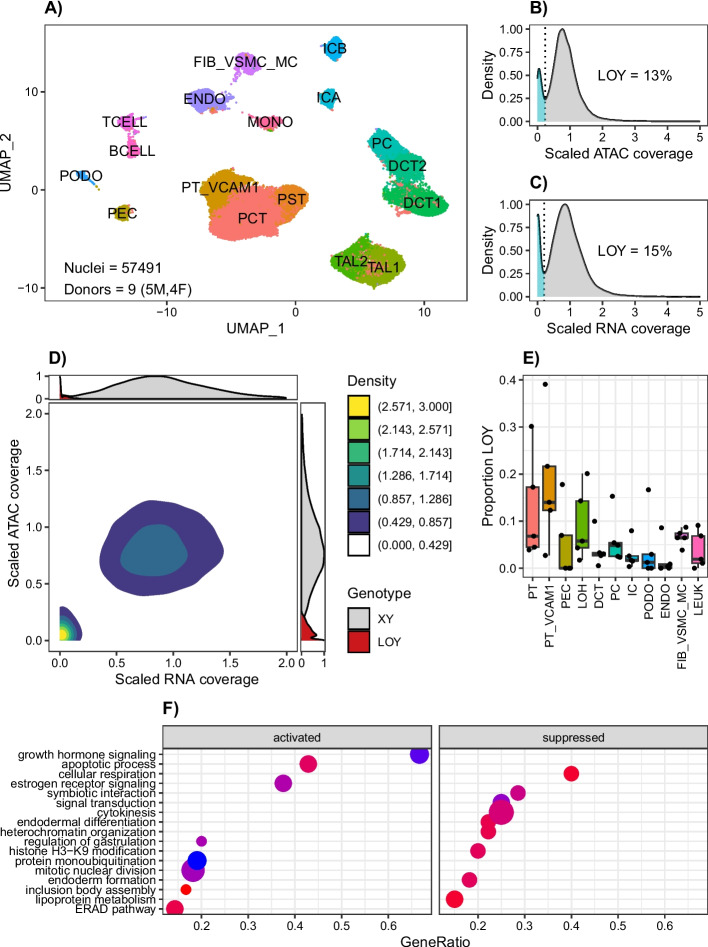
LOY detection by single nucleus multiome sequencing. **A** UMAP of male and female kidney cell types. PCT, proximal convoluted tubule; PST, proximal straight tubule; PT_VCAM1, VCAM1 + proximal tubule; PEC, parietal epithelial cells; TAL1, cortical thick ascending limb; TAL2, medullary thick ascending limb; DCT1, early distal convoluted tubule; DCT2, late distal convoluted tubule; PC, principal cells; ICA, type A intercalated cells; ICB, type B intercalated cells; PODO, podocytes; ENDO, endothelial cells; FIB_VSMC_MC, fibroblasts; vascular smooth muscle; and mesangial cells; TCELL, T cells; BCELL, B cells; MONO, mononuclear cells. **B** ATAC modality LOY density plot in 23,333 male cells. **C** RNA modality LOY density plot in 23,333 male cells **D** Joint ATAC/RNA LOY density plot in 23,333 male cells. **E** Median proportion LOY in male kidney cell types. **F** Gene ontology pathway enrichment for age-adjusted differentially expressed genes associated with LOY in the male proximal tubule

We prepared six single nucleus multiomes (10X Genomics, Single Cell Multiome ATAC + Gene Expression) from adult kidney cortex and aggregated them with three previously published multiomes to simultaneously measure gene expression and chromatin accessibility in every cell [21]. Samples were obtained from control donors or donors with CKD and included both men (*n* = 5) and women (*n* = 4, Additional File 1: Clinical Data and Quality Control). In total, our dataset had 57,491 male and female nuclei after removal of doublets and low-quality barcodes (Fig. 1A, Additional File 2: Fig S1). All major cell types in the kidney cortex were represented, including a population of *VCAM1* + proximal tubule cells (PT_VCAM1). PT_VCAM1 is an injured cell state driven by NFkB signaling that is detectable in histologically normal kidney [22]. Its proportion increases in aging and CKD and we hypothesize that its pro-inflammatory expression and chromatin accessibility profile is due in part to DNA damage [23].

To evaluate cells for LOY, we quantified single-cell Y chromosome RNA transcript counts or ATAC fragments and visualized the density estimate for both modalities. To control the false positive rate, we limited our analysis to the subset of nuclei with at least 10,000 ATAC fragments and 1,000 RNA transcript counts. As expected, female samples had a negligible amount of RNA transcripts and ATAC fragments mapping to the Y chromosome (Additional File 2: Fig S2A, S2B). In contrast, male samples had a bimodal distribution—where a minority of cells had no detectable Y chromosome RNA transcripts or ATAC fragments (Fig. 1B,C). We used the trough of the density estimate as a threshold to quantify the proportion of male cells with LOY and obtained similar results for RNA (*N* = 3386/23333 = 15%) and ATAC (*N* = 3161/23333 = 13%) modalities. The estimated proportion of cells with LOY varied by donor but was independent of sequencing depth, suggesting that LOY is not a result of inadequate sampling (Additional File 2: Fig S2C, S2D). Moreover, the median Y chromosome coverage had limited variation between cell types, which means that LOY determination is not significantly affected by cell-specific chromatin remodeling. The distribution of other chromosomes was approximately normal (Additional File 2: Fig S3), which is consistent with reports that LOY is by far the most common structural variant in peripheral blood of aging men [24]. To confirm our findings, we visualized both modalities together to show that male cells without Y chromosome transcripts also lack Y chromosome ATAC fragments (Fig. 1D). We used a finite mixture model to incorporate both modalities and make a joint estimate of the total proportion of LOY (*N* = 2626/23333 = 11%), which was slightly more conservative than our unimodal estimates.

There are more than twenty cell types in the kidney and each of them responds differently to injury. The proximal tubule is the most abundant cell type in the cortex and can undergo de-differentiation and division following ischemia reperfusion [17, 25]. In contrast, podocytes are terminally differentiated cells that do not have the ability to divide. Somatic mutations arise in tissues at different rates, and we hypothesized that LOY would occur in kidney cell types at different rates—reflecting a marker of DNA damage and cellular stress [26]. We used the genotypes from our finite mixture model to estimate the proportion of LOY for kidney cell types (Fig. 1E). In general, cortical epithelial cell types (PT, PT_VCAM1, PEC, LOH) had a higher proportion of LOY than medullary epithelial cell types (DCT, PC, IC), podocytes, and endothelial cells. We used a generalized linear model with a mixed effect (GLMM) per donor to estimate the odds of observing LOY in each cell type relative to the proximal tubule (Additional File 2: Fig S4). Among all cell types, PT_VCAM1 had the greatest likelihood for LOY compared to PT (OR = 1.46, 95% CI [1.29–1.65], *p* = 1.1e − 09), whereas podocytes had a much lower likelihood (OR = 0.17, 95% CI [0.07–0.40], *p* = 3.5e − 05). The thick ascending limb had a similar likelihood for LOY compared to PT (OR = 1.05, 95% CI [0.94–1.17], *p* = 0.3).

We next compared the transcriptional profile of cells without a Y chromosome (LOY) to cells with a Y chromosome to determine if there are cell-specific differentially expressed genes associated with LOY (Additional File 3). The majority of differentially expressed genes were in the proximal convoluted tubule (PCT^LOY^ vs. PCT^XY^, *n* = 218, padj < 0.05) and cortical thick ascending limb (TAL1^LOY^ vs. TAL1^XY^, *n* = 213, padj < 0.05), which were also the cell types with the greatest number of LOY cells. Because of the close association between LOY and age, we adjusted our differential expression analysis for donor age (Additional File 4). After age-adjustment, the proximal tubule had an even greater number of differentially expressed genes (PCT^LOY^ vs. PCT^XY^, *n* = 321, padj < 0.05), and approximately one third of these genes were shared between adjusted and unadjusted analyses (*n* = 103). We performed a gene set enrichment analysis using the age-adjusted gene list to further explore pathways associated with LOY in the proximal tubule. PCT^LOY^ showed activation of pathways involved in cell division and suppression of pathways involved in chromatin assembly (Fig. 1F).

LOY cells also had a different chromatin accessibility profile (Additional File 5). Aside from the Y chromosome, there were a modest number of differentially accessible peaks across multiple cell types. Many of these peaks were located near X chromosome genes and associated with decreased accessibility (*ASMTL*, *SLC25A6*, *AKAP17A*, *GTPBP6*, *PPP2R3B*, *CD99*, *GTPBP6*, *DMD*). There was also evidence of differential transcription factor activity (Additional File 6). The majority of transcription factors with differential activity were located in the proximal tubule and thick ascending limb. HNF1A and HNF1B were two transcription factors with significantly decreased activity in proximal tubule cells with LOY.

### LOY detection by single nucleus ATAC sequencing

**Fig. 2:**
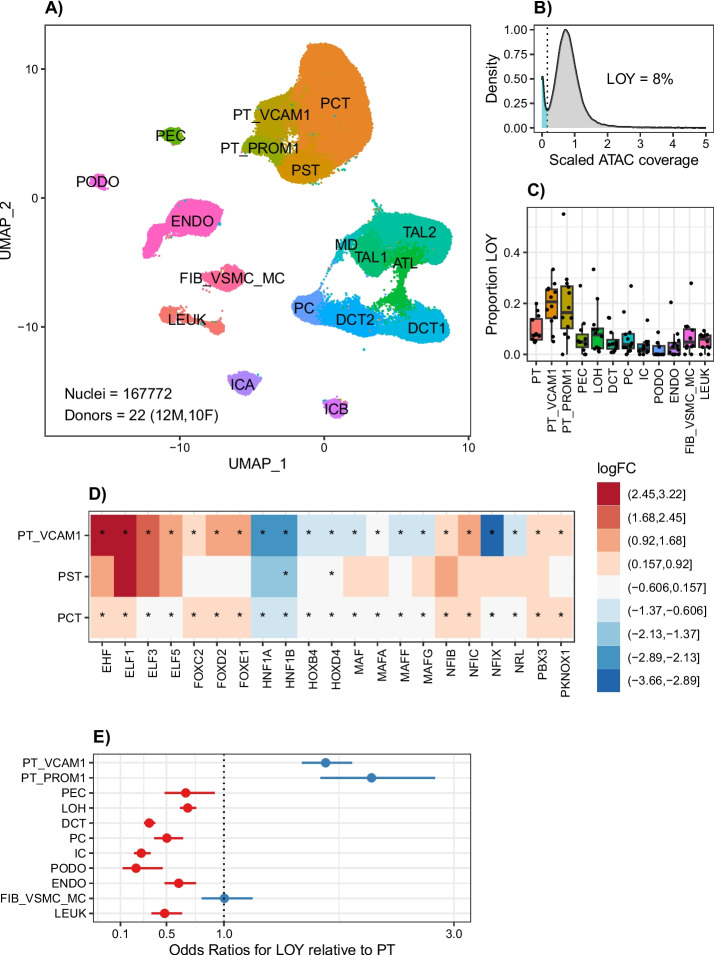
snATAC-seq detection of LOY. **A** UMAP of male and female kidney cell types PCT, proximal convoluted tubule; PST, proximal straight tubule; PT_VCAM1, VCAM1 + proximal tubule; PT_PROM1, PROM1 + proximal tubule; PEC, parietal epithelial cells; ATL, ascending thin limb; TAL1, cortical thick ascending limb; TAL2, medullary thick ascending limb; MD, macula densa; DCT1, early distal convoluted tubule; DCT2, late distal convoluted tubule; PC, principal cells; ICA, type A intercalated cells; ICB, type B intercalated cells; PODO, podocytes; ENDO, endothelial cells; FIB_VSMC_MC, fibroblasts, vascular smooth muscle, and mesangial cells; LEUK*—*leukocytes. **B** ATAC modality LOY density plot in 47,458 male cells. **C** Median proportion LOY in male kidney cell types. **D** Log-fold-change transcription factor activity for LOY vs. XY in male kidney cell types for transcription factors with differential activity in at least two proximal tubule subpopulations. **F** GLMM for LOY in male kidney cell types

We prepared five single nucleus ATAC (snATAC-seq) libraries from donors with CKD and aggregated them with previously published snATAC-seq data from control and CKD donors to create an integrated atlas (Fig. 2A). In total, we analyzed 167,772 nuclei from 22 donors, including both men (*n* = 12) and women (*n* = 10). One advantage of analyzing a large snATAC-seq atlas is its improved ability to detect proximal tubule subpopulations. We identified multiple populations of injured proximal tubule cells that we termed PT_VCAM1 and PT_PROM1. PT_VCAM1 is *PROM1-VCAM1* + , which is what differentiates it from PT_PROM1, which is *PROM1* + *VCAM1-* (Additional File 2: Fig S5). *PROM1* encodes CD133, which is a marker of cancer stem cells and de-differentiation [27].

We evaluated male cells for LOY by estimating the density of Y chromosome ATAC fragments and observed a similar bimodal distribution (Fig. 2B). In total, 8% of snATAC-seq nuclei had LOY and cortical epithelial cell types (PT, PT_VCAM1, PT_PROM1, PEC, LOH) had a greater proportion of LOY than medullary epithelial cell types (DCT, PC, IC) and podocytes. Importantly, the proportion of LOY was not dependent on sequencing depth, which excludes the possibility that LOY is a result of inadequate sampling (Additional File 2: Fig S6). Strikingly, PT_VCAM1 and PT_PROM1 were the two cell types with the highest proportion of LOY (Fig. 2C). Much like the single-cell multiomes, LOY cells showed decreased accessibility of peaks located near X chromosome genes in the proximal tubule (*ASMTL*, *CD99*, *SLC25A6*, *GTPBP6*, Additional File 7).

LOY was also associated with cell-specific differential transcription factor activity in the snATAC-seq dataset (Additional File 8). The majority of transcription factors that showed differential activity were in the proximal tubule, and a subset of them were identified in multiple proximal tubule populations (Fig. 2D). HNF1A and HNF1B are two transcription factors that are closely associated with kidney disease, and both of these transcription factors showed decreased activity in proximal tubule cells with LOY [28, 29]. HNF1A is upregulated during proximal tubule differentiation from human pluripotent stem cell-derived kidney organoids, and we hypothesize that HNF1A activity is reflective of a de-differentiated state in cells with LOY [30]. We used our GLMM to estimate the odds of observing LOY in each cell type relative to the proximal tubule. Both PT_VCAM1 (Fig. 2E, OR = 1.88, 95% CI [1.67–2.11], *p* = 6.5e − 27) and PT_PROM1 (Fig. 2E, OR = 2.28, 95%CI [1.83–2.83], *p* = 8.9e − 14) had a greater likelihood for LOY compared to PT.

LOY is well-described in circulating leukocytes, but leukocytes are underrepresented in single-cell datasets in the kidney. To determine the relative proportion of leukocytes with LOY in the peripheral blood and kidney, we reanalyzed publicly available snATAC-seq data from donors with renal cell carcinoma [31]. These data used CD45-enrichment to increase the number of leukocytes obtained from matched peripheral blood (*n* = 8), tumor-adjacent (*n* = 4), and renal carcinoma (*n* = 8) samples from eight donors. The donors included seven men and one woman and ranged in age from 45 to 79. The mean age of the male donors was 57 years. We aggregated all of the CD45-enriched snATAC-seq libraries and obtained 61,146 nuclei after quality control (Additional File 2: Fig S7). We annotated the cells using bridge integration with a publicly available leukocyte multiome atlas [32]. The dataset consisted of predominantly T cells (67%), B cells (12%), and NK cells (12%) with smaller proportions of monocytes and dendritic cells. Approximately 1% of male CD45 + leukocytes had LOY (738/51898) and the majority of cells with LOY were B or T cells. The proportion of leukocytes with LOY in the CD45-enriched datasets was somewhat lower than the kidney snATAC-seq dataset (5.4%). These differences may be due to a younger age in the CD45-enriched male donors (57 vs 62y) or variable cellular composition.

### LOY detection by single-cell and single nucleus RNA sequencing

**Fig. 3:**
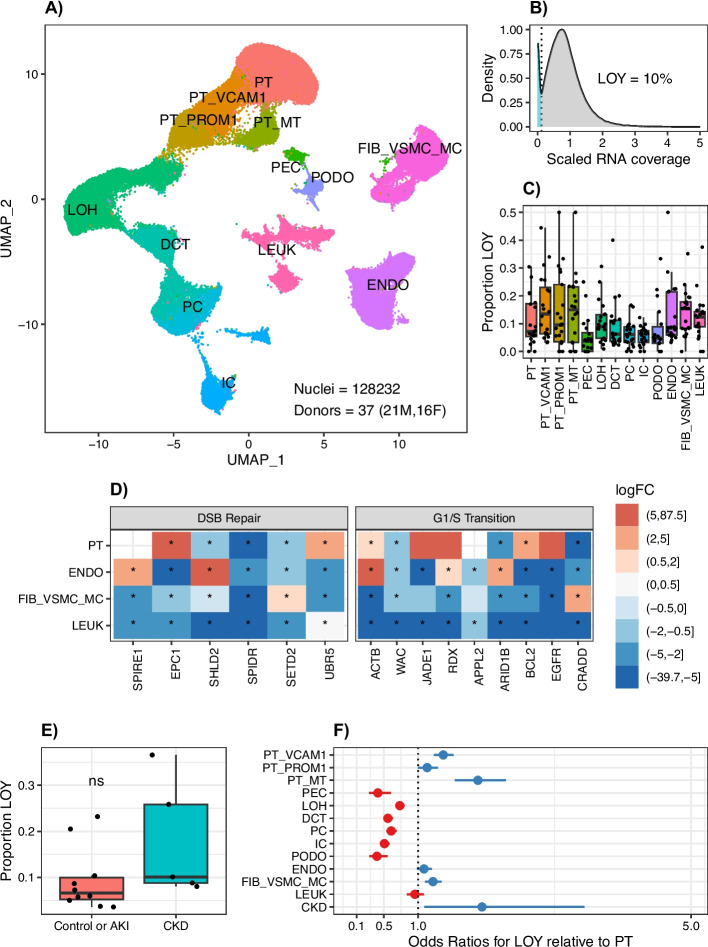
LOY detection by scRNA-seq. **A** UMAP of male and female kidney cell types PT, proximal tubule; PT_VCAM1, VCAM1 + proximal tubule; PT_PROM1, PROM1 + proximal tubule; PT_MT, mitochondrial gene proximal tubule; PEC, parietal epithelial cells; LOH, loop of Henle; DCT, distal convoluted tubule; PC, principal cells; IC, intercalated cells; PODO, podocytes; ENDO, endothelial cells; FIB_VSMC_MC, fibroblasts; vascular smooth muscle, and mesangial cells, LEUK*—*leukocytes. **B** RNA modality LOY density plot in 78,738 male cells. **C** Median proportion LOY in male kidney cell types. **D** Log-fold-change gene expression for LOY vs. XY in male kidney cell types. **E** Proportion LOY in Control or AKI vs CKD male samples. **F** GLMM for LOY in male kidney cell types adjusted for CKD

We reanalyzed a publicly available scRNA-seq/snRNA-seq atlas of adult human kidney from the Kidney Precision Medicine Project (KPMP). The KPMP dataset has 37 donors, including men (*n* = 21) and women (*n* = 16), with samples obtained from control donors, donors with acute kidney injury (AKI), and donors with CKD across a wide range of ages. In total, we analyzed 128,232 male and female cells, which represented all of the major cell types in the kidney cortex (Fig. 3A, Additional File 2: Fig S8). We detected three populations of injured proximal tubule cells that we termed PT_VCAM1, PT_PROM1, and PT_MT. PT_VCAM1 cells expressed *HAVCR1*, *VCAM1*, and *BCL2*. PT_PROM1 cells expressed *PROM1*, *TNIK*, and *TNFSF10*. Our PT_VCAM1 and PT_PROM1 annotations were highly overlapping with adaptive proximal tubule cells (aPT), which were predictive of future kidney function decline in the KPMP publication [19]. The PT_MT population expressed oxidative phosphorylation and apoptosis pathway genes and was highly overlapping with the KPMP degenerative proximal tubule cell (dPT) annotation. We evaluated male cells for LOY by estimating the density of Y chromosome RNA counts and detected 10% of cells with LOY (Fig. 3B). Similar to our single-cell multiome and snATAC-seq analysis, the injured proximal tubule subsets (PT_VCAM1, PT_PROM1, PT_MT) had a greater proportion of LOY compared to proximal tubule, thick ascending limb, distal nephron, and glomerular cell types (Fig. 3C). Intriguingly, the KPMP dataset had a greater number of endothelial cells, fibroblasts, and infiltrating leukocytes with LOY. Leukocytes with LOY have been reported to traffic to the heart and kidney where they promote fibrosis [33].

We next compared the transcriptional profile of cells without a Y chromosome (LOY) to cells with a Y chromosome to determine if there are cell-specific differentially expressed genes associated with LOY in the KPMP dataset (Additional File 9). We identified differentially expressed genes (DEG) across all cell types, but the cell types with the largest number of DEG included PT (PT^LOY^ vs. PT^XY^, *n* = 3333, padj < 0.05) and DCT (DCT^LOY^ vs. DCT^XY^, *n* = 4062, padj < 0.05). PT^LOY^ showed enrichment for pathways involved in cellular response to DNA damage and G1/S transition (Fig. 3D). In addition, PT^LOY^ had increased expression of pro-survival genes like *BCL2*. In the proximal tubule, the majority of differentially expressed genes were retained after age-adjustment (Additional File 10, *n* = 2265), and these genes were enriched for pathways involved in apoptosis, cell transport, and differentiation among others. We identified many of the same pathways when we compared PT_VCAM1^LOY^ to PT_VCAM1^XY^ and PT_PROM1^LOY^ to PT_PROM1^XY^ (Additional File 2: Fig S9). DNA repair and cell cycle genes are two pathways that have been associated with LOY. LOY was associated with decreased expression of DNA repair and cell cycle genes across multiple cell types—suggesting that LOY is associated with an injury response in multiple cell states (Additional File 2: Fig S10). Although this is not direct evidence of the proliferative history of these cells, it does point to a senescent phenotype and decreased likelihood for cell division.

We used the KPMP clinical meta data to compare donors with CKD to donors without CKD and there was a trend towards greater proportion of LOY in CKD vs. control and AKI samples (control or AKI: LOY = 0.08 ± 0.06, CKD: LOY = 0.17 ± 0.06, Wilcoxon *p* = 0.055). To further evaluate the association between CKD and LOY, we added CKD as a predictor variable to our GLMM and estimated the odds of observing LOY in each cell type relative to the proximal tubule. CKD was associated with a greater likelihood for LOY (Fig. 3E, OR = 1.92, 95% CI [1.06–3.46], *p* = 0.03) after adjusting for cell type. In addition, PT_VCAM1 (Fig. 3E, OR = 1.87, 95% CI [1.67–2.10], *p* = 6.9e − 27), PT_PROM1 (Fig. 2E, OR = 1.37, 95% CI [1.22–1.53], *p* = 2.0e − 08), and PT_MT (Fig. 3E, OR = 2.05, 95% CI [1.67–2.50], *p* = 1.7e − 12) were among the cell types with the highest odds for LOY after adjusting for CKD.

### LOY detection by digital PCR

**Fig. 4:**
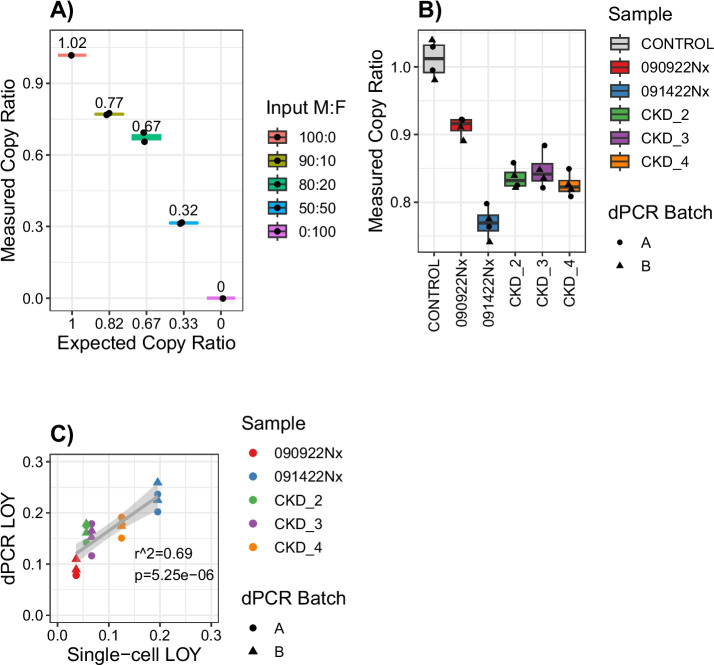
LOY detection by digital PCR. **A** gDNA was extracted from male hTERT-RPTEC or female HEK293T cell lines and mixed in the indicated male: female (M:F) ratios. The mixed DNA samples were measured by multiplex dPCR using a FAM-labeled chrY target and ABY-labeled chrX reference. The measured copy ratio is the number of microwells that amplify the chrY target divided by the number of microwells that amplify the chrX reference. These values were compared to the expected copy ratio, which was estimated based on the relative ratio of M:F DNA input. For example, a 50:50 mixture of M:F DNA would yield 1 copy of chrY for every 3 copies of chrX and an expected copy ratio of 0.33. **B** gDNA was extracted from male kidney cortex samples and assayed by dPCR as previously described. Each sample was assayed with 2 technical replicates and 2 independent batches for a total of 4 measurements per sample. The sample labeled control is gDNA isolated from a male hTERT-RPTEC cell line. **C** We used dPCR to estimate the fraction of male cells with LOY using the following formula: 1 − chrY/chrX. This approach assumes the concentration of chrX is constant and that any changes in the measured copy ratio are due to changes in the concentration of chrY. We compared our dPCR and single-cell estimates to compute an r-squared value using the lm function in R

Single-cell sequencing is sparse, which makes it difficult to distinguish cells with low Y chromosome expression or accessibility from cells with LOY. Digital PCR (dPCR) is a sensitive method for quantifying copy number variation that has been previously used to measure LOY in blood [34]. We used a similar approach to design a multiplex dPCR assay and estimate the proportion of cells with LOY in the kidney. Our instrument uses microfluidics to disperse DNA into 20,000 microwells for each sample. Each microwell undergoes end-point PCR using a target probe on the Y chromosome and a reference probe on the X chromosome. Subsequently, we count the number of microwells that amplify each chromosome and compute a ratio. In a euploid male tissue, the number of X and Y chromosomes should be equal, but as the proportion of cells with LOY increases, the ratio of Y chromosome to X chromosome will decrease. To validate our assay, we simulated LOY by varying the ratio of male to female DNA isolated from euploid cell lines. As the proportion of female DNA increases, our dPCR assay measures a decreased ratio of chrY:chrX (Fig. 4A). The measured copy ratio for each data point was within 5% of the expected copy ratio and the technical replicates had a coefficient of variation on the order of 1–2%. These data suggest that dPCR is sufficiently sensitive to detect LOY in the range observed in our human samples.

We isolated gDNA from a nearby region of the same kidneys that were used to prepare single-cell libraries and performed dPCR. These samples included two male multiomes and three male snATAC-seq libraries. All of these samples showed a significant reduction in the chrY:chrX ratio compared to the euploid control (Fig. 4B)—suggesting that LOY in the kidney is measurable by dPCR. In addition, the measured copy ratios were consistent between technical replicates and independent batches, which points to the reproducibility of our assay. Next, we compared dPCR and single-cell LOY estimates to determine if these methods yield similar results when measuring independent samples from the same donor. There was a significant positive correlation between the dPCR and single-cell estimates, which suggests that both methods can measure LOY (Fig. 4C).

### Spatial analysis and intercellular signaling

**Fig. 5:**
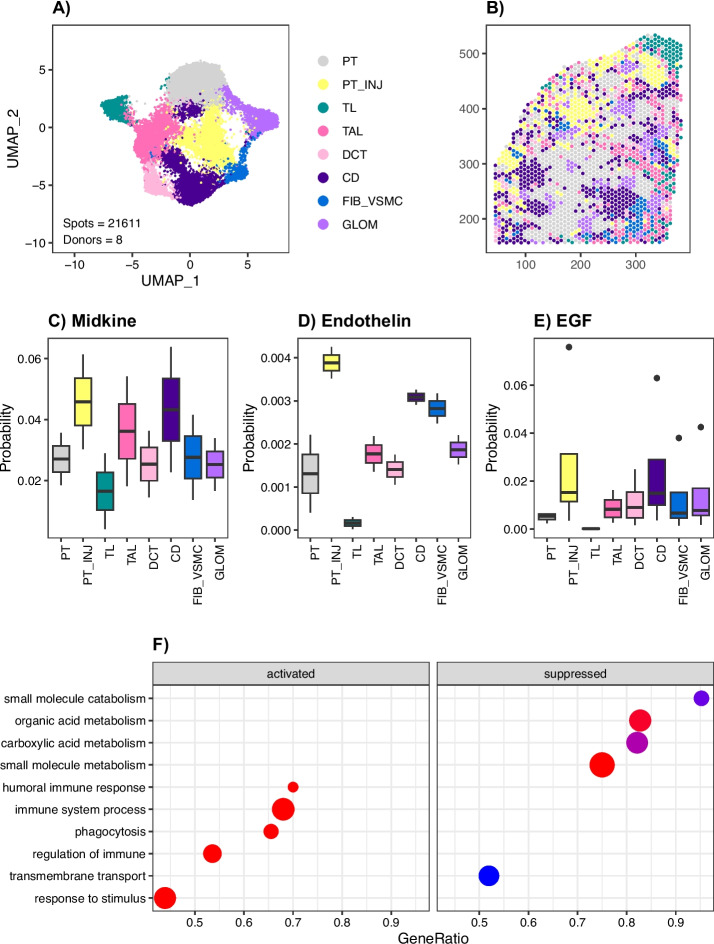
Spatial transcriptomics in the kidney identifies a pro-fibrotic microenvironment. **A** UMAP of integrated spatial datasets with neighborhood annotations PT, proximal tubule; PT_INJ, injured proximal tubule; TL, thin limb; TAL, thick ascending limb; DCT, distal convoluted tubule; CD, collecting duct; FIB_VSMC, fibroblasts and vascular smooth muscle cells; GLOM, glomeruli. **B** Representative spot annotation and visualization of spatial neighborhoods. **C** CellChat midkine secreted signaling arising from the PT_INJ neighborhood. **D** CellChat endothelin secreted signaling arising from the PT_INJ neighborhood. **E** CellChat EGF secreted signaling arising from the PT_INJ neighborhood. **F** Gene ontology enrichment for PT_INJ spot-specific differentially expressed genes

We prepared eight spatial transcriptomic libraries (10X Visium) from donors with and without CKD to examine intercellular signaling patterns in the kidney across 21,611 spots. Four of the eight spatial libraries came from male donors. In this method, there are multiple cells per 55uM spot and multiple spots compose a neighborhood of cells. We integrated the spatial datasets and annotated spots using lineage-specific markers (Fig. 5A). In doing so, we were able to identify neighborhoods that roughly correspond to different cell types in the kidney, including the proximal tubule (PT), loop of Henle (LOH), glomeruli (GLOM), fibroblasts and vascular smooth muscle (FIB_VSMC), and collecting duct (CD). We also identified a neighborhood of injured proximal tubule cells that was admixed with leukocytes (PT_INJ). PT_INJ spots expressed *VCAM1*, *PROM1*, and *HAVCR1* much like PT_VCAM1 and PT_PROM1 cells in our single-cell datasets (Additional File 2: Fig S11). A representative image shows that most spots correspond to the proximal tubule, but a subset are annotated as PT_INJ (Fig. 5B). Although our spatial dataset is not a single-cell resolution, it can provide insight into intercellular signaling patterns by quantifying secreted ligand-receptor interactions between adjacent spots. We were especially interested in the intercellular signals that arise from injured proximal tubule cells (PT_INJ)—some of which may be communicating with nearby leukocytes.

Newer methods for ligand-receptor analysis incorporate distance constraints that penalize long-range intercellular signaling [35]. This approach helps to prioritize secreted signaling interactions that preferentially occur within tissue microenvironments. We used CellChat to measure ligand-receptor interactions arising from the PT_INJ neighborhood. Some of the upregulated signaling pathways included midkine, endothelin, and EGF signaling (Fig. 5C–E). Each of these pathways has been previously implicated in CKD progression, which suggests that the PT_INJ neighborhood may be acting as a pro-fibrotic microenvironment that secretes autocrine and paracrine cytokines [36–38]. To further explore this hypothesis, we used Seurat to identify neighborhood-specific genes and performed GSEA to show that the PT_INJ neighborhood has activation of immune-related signaling pathways (Fig. 5F).

### Age and mosaic chromosomal alterations are associated with LOY in the kidney

**Fig. 6:**
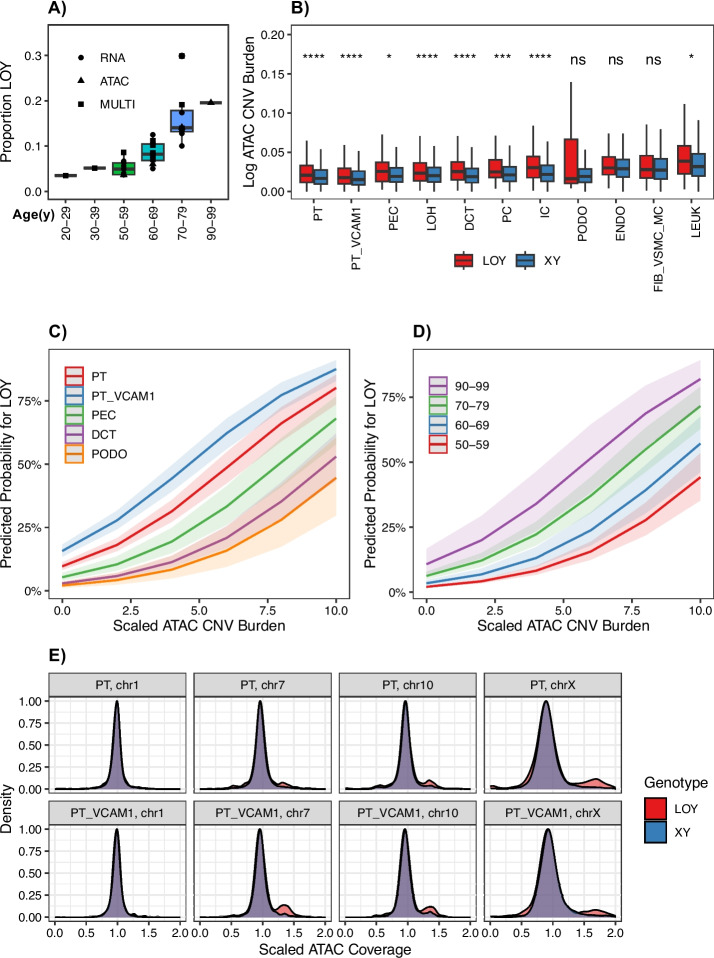
Age and mCA as Predictors for LOY. **A** Proportion LOY for single-cell multiomes, snATAC-seq and scRNA-seq for male donors (*n* = 32) binned by age. **B** Genome-wide CNV burden for LOY vs XY in male single-cell multiomes and snATAC-seq datasets. **C** Marginal probabilities for LOY by cell type and CNV burden in male single-cell multiomes and snATAC-seq. **D** Marginal probabilities for LOY by age and CNV burden in male single-cell multiomes and snATAC-seq. **E** Coverage density for male kidney cell types by chromosome in LOY and XY cells

Age is a major risk factor for LOY in the blood and we hypothesized that age may also play a role in the kidney. We estimated total proportion of LOY for all male donors (*n* = 32) using the single-cell multiome, snATAC-seq, and KPMP datasets and binned the estimates by donor age (Fig. 6A). There was a clear positive correlation between LOY and donor age (Pearson *r*^2^ = 0.70, *p* = 2.3e − 05), suggesting that age is a risk factor for LOY in the kidney.

Although LOY is the most common structural variant in men, there are a multitude of mosaic chromosomal alterations (mCA) that occur in somatic tissues [39]. In peripheral blood, men develop more mCA than women, which is largely driven by gain events [40]. LOY in murine hematopoietic progenitors promotes DNA damage and leukemogenesis, which suggests that LOY acts as both a marker of DNA damage and a risk factor for additional DNA damage [41]. In support of this hypothesis, LOY is often seen together with additional somatic mutations, like whole chromosome gains, in human non-neoplastic kidney tissue [11]. To evaluate the association between LOY and mCA in the kidney, we used epiAneufinder to call single-cell somatic CNV in 1 MB bins across autosomal chromosomes to estimate genome-wide CNV burden in single-cell multiomes and snATAC-seq datasets [42]. Strikingly, CNV burden was increased in LOY cells compared to cells with a Y chromosome (XY) in a majority of cell types (Fig. 6B).

We incorporated cell type, age, and CNV burden into our GLMM and each of these variables was a significant predictor of LOY (Additional File 2: Fig S12). To visualize our results, we estimated the marginal effects for LOY. The predicted probability for LOY was higher in the proximal tubule (PT, PT_VCAM1) compared to the distal nephron and podocytes across the range of CNV burden (Fig. 6C, Additional File 2: Fig S13). Similarly, the probability for LOY was higher in older individuals and increased along with CNV burden (Fig. 6D), which is consistent with accumulation of mutations in aging. We hypothesized that increased CNV burden associated with LOY may be due to large chromosomal gains and focused on the proximal tubule (PT, PT_VCAM1) to visualize differences between LOY and cells with an XY genotype. The majority of chromosomes, like chromosome 1, showed no difference between LOY and cells with an XY genotype (Fig. 6E). This may be because mCA involving chromosome 1 are uncommon, or alternatively, cells with these alterations are more likely to undergo apoptosis. In contrast, a subset of LOY cells showed increased coverage suggestive of gains on chromosomes 7, 10, and X (Fig. 6E). Selective gains of chromosomes 7 and 10 are among the most frequent CNV in cell culture models of the proximal tubule and are often seen in renal tumors [43]. We observed increased chromosome 7 CNV burden in LOY compared to XY cells for both PT (fold change = 1.23, Wilcoxon rank sum with Bonferroni padj = 8.3e − 12) and PT_VCAM1 (fold change = 1.48, Wilcoxon rank sum with Bonferroni padj = 1.9e − 03). Trisomy 7 is a frequent occurrence in renal tumors and is associated with increased *EGFR* expression, which suggests that these CNV may confer a survival advantage [44]. Furthermore, LOY is associated with gain of the X chromosome in approximately half of male cancer cell lines and may confer increased cellular fitness [45].

We performed a joint analysis of the multiome and KPMP datasets to determine which genes are differentially expressed due to LOY. Across all cell types, we identified 2235 differentially expressed genes that met the age-adjusted *p*-value threshold (Additional File 11). Among the 182 DEGs in the PCT, the pathways were enriched for mitochondrial electron transport and cytoplasmic translation. Some of these same pathways were enriched in B cells and mononuclear cells with LOY.

## Discussion

Somatic mosaicism is increasingly recognized as an important regulator of tissue biology [2]. LOY is one of the most well-characterized mCA because it is large (~ 60 Mb) and relatively easy to detect in peripheral blood. For many years, LOY was thought to be part of normal male aging, but recent studies have shown that the Y chromosome plays a role beyond male development [46]. These functions may partially explain sex differences in disease and cancer predisposition [47]. The mechanism through which LOY occurs is unknown, but the Y chromosome has a unique centromere structure that may lead to chromosome mis-segregation during mitosis [48]. In this model, the more times a cell divides, the more likely it is to experience LOY. As a result, highly proliferative cell types may be particularly susceptible to LOY.

Many of the studies on LOY are focused on leukocytes [49–52]. LOY is related to clonal hematopoiesis of indeterminate potential (CHIP), which is defined by the presence of clonally expanded hematopoietic cells that share somatic mutations but do not meet criteria for neoplasia. CHIP has been associated with a growing list of conditions, including age, smoking, heart disease, and kidney disease [53]. Leukocytes with LOY have a unique transcriptional signature and decreased expression of CD99, which is an important regulator of migration, differentiation, and apoptosis [49]. LOY may also affect leukocyte subsets differently. Men with Alzheimer’s disease develop LOY in NK cells whereas men with prostate cancer were more likely to develop LOY in CD4 + T cells and granulocytes [50].

LOY has also been detected in other human tissues. Single-cell sequencing has shown LOY in human brain-derived microglia and blood-derived myeloid cells [7]. LOY led to a transcriptional signature associated with aging and inflammation and microglia have been implicated in the progression of Alzheimer’s disease [7]. LOY is also known to play an important role in cancer [10]. In the bladder, LOY tumors had a worse prognosis which may be related to their ability to evade the immune system. Strikingly, LOY tumors had an increased response to anti-PD-1 checkpoint inhibitors, which may point towards the prognostic value of LOY [10].

The mechanism through which LOY promotes disease progression is unknown, but animal models have provided some insight. Deletion of the Y chromosome from hematopoietic progenitors leads to cardiac fibrosis, kidney fibrosis, short-term working memory deficits, and increased mortality in mice [33]. Macrophages with LOY traffic to the heart where they adopt a pro-fibrotic phenotype that is mitigated by a TGFB1-neutralizing antibody. These data show that LOY in leukocytes can accelerate tissue fibrosis and raise the question whether LOY in other cell types exerts a similar effect. There is evidence from animal models that the rate of Y chromosome loss is tissue-dependent. By 2 years of age, rats develop LOY in the blood, brain, kidney, testis, and liver, but they do not develop LOY in the lung, muscle, pancreas, skin, or spleen [54]. Importantly, this study used qPCR of bulk tissue, which may miss LOY in underrepresented cell types.

CKD is characterized by a progressive decline in kidney function associated with increased fibrosis [16]. Single-cell sequencing has identified a subset of proximal tubule cells with a pro-inflammatory signature that predicts future kidney function decline. These cells variably express *VCAM1*, *PROM1*, *CD24*, and *HAVCR1* and have been called a variety of things in the literature, including PT_VCAM1/PT_PROM1, scattered tubular cells, adaptive/maladaptive proximal tubule cells, failed-repair proximal tubule, and injured proximal tubule [19, 22, 23, 55–57]. Here, we use single-cell sequencing to show that these cells are more likely to have LOY than other kidney cell types. Moreover, age is a risk factor for LOY in the kidney and kidney cells with LOY have increased genomic instability associated with expression of pro-survival pathways. These alterations may increase the risk for tumor formation; and in fact, the *VCAM1* + proximal tubule is transcriptionally related to renal cell carcinoma [58]. However, this cell state is not limited to male kidneys—so what is happening in female cells? We hypothesize that female samples also have increased DNA damage but do not have a signal that is as easy to detect as LOY. One potential explanation is mosaic loss of X chromosome (LOX). LOX is associated with female aging but occurs at a much lower rate than LOY [59]. We did not find enrichment for LOX in female cells, but LOX typically affects the inactive X chromosome, which is not actively transcribed and may be difficult to detect with our approach. Additional alterations not measured in this study include copy-neutral loss-of-heterozygosity, telomere length, and other hallmarks of cellular senescence. Sexual dimorphism plays an important role in transcriptional regulation of the proximal tubule [60]. In particular, androgens are important regulators of salt reabsorption and water volume in men. It is possible that some of the differences between the male and female kidney are due to Y chromosome biology. However, whether LOY or increased susceptibility to DNA damage contributes to the increased incidence of kidney cancer in men is unclear [61]. In our analysis, LOY cells had a transcriptional signature consistent with DNA damage and cellular senescence whether they were compared to male or female cells. These data suggest that LOY, and DNA damage in general, may exert a conserved transcriptional response independent of cell type and sex.

Single-cell sequencing is sparse and may underestimate the true burden of mCA. Large mCA like LOY are relatively easy to find provided there is adequate sequencing depth. However, smaller mCA may not be adequately sampled and are undetectable by single-cell sequencing. snRNA-seq has somewhat lower sensitivity because it can only detect mCA that result in changes in transcript abundance and it is subject to technical noise arising from biological variability, ambient RNA contamination, and ambiguously mapped reads. This is a significant limitation because the majority of the genome is not transcribed and an unknown proportion of mCA (e.g., copy-neutral LOH) may not alter transcript abundance. snATAC-seq has more uniform coverage compared to snRNA-seq but is still restricted to areas of accessible chromatin. It also suffers from sparsity and has limited ability to detect small mCA. For these reasons, it will be necessary to have complementary assays that can validate the presence or absence of mCA in a tissue sample. Here, we developed a multiplex dPCR assay to quantify LOY. Some of the advantages of this assay include higher throughput and lower cost, which may make it useful as a screening or diagnostic assay. Future studies may explore whether the proportion of cells with LOY is associated with CKD stage or if LOY has prognostic value for CKD progression. Alternatively, targeted therapies might seek to eliminate LOY cells, and this assay could be used to evaluate efficacy. Therapies have been developed to target senescent cells and prevent CKD progression after acute kidney injury; however, it remains unclear whether similar therapies might be used to target cells with mCA [62].

## Conclusions

This study demonstrates LOY in the kidney by single-cell sequencing and digital PCR. The proportion of each cell type with LOY varied depending on its location in the nephron and injured proximal tubule cells had the highest proportion of LOY. We hypothesize that LOY is an indicator of increased DNA damage and potential marker of cellular senescence that can be applied to single-cell datasets in other tissues.

## Methods

### Human kidney tissue

Tissue samples prepared for this publication include single nucleus multiomes (*n* = 6), snATAC-seq (*n* = 5) and Visium spatial datasets (*n* = 8). Tissue samples were obtained from non-tumor kidney cortex from deceased organ donors or patients undergoing nephrectomy at Washington University in St. Louis (St. Louis, MO, USA). All participants provided written informed consent in accordance with the Declaration of Helsinki, including publication of demographic and clinical history as included in Additional File 1. Histologic sections were reviewed by a renal pathologist and laboratory data was abstracted from the medical record. CKD was determined based on eGFR less than 60 mL/min/1.73 m^2^ using KDIGO guidelines [63].

### Statistics and reproducibility

Statistical analysis was conducted on all collected samples and analysis was done in Docker containers to enhance reproducibility. The details of each analysis are outlined in the methods section and all of the code has been made publicly available on GitHub. No statistical method was used to predetermine sample size. No data were excluded from the analyses. The experiments were not randomized. Investigators were not blinded to allocation during experiments and outcome assessment.

### Nuclear dissociation and library preparation

For single nucleus multiomes, samples were cut into < 2 mm pieces and homogenized using a Dounce homogenizer with the loose head pestle (885,302–0002; Kimble Chase) in 2 ml of Nuclei EZ Lysis buffer (NUC-101; Sigma-Aldrich) with protease inhibitor (5,892,791,001; Roche) and Protector RNase Inhibitor (Roche, 3,335,402,001; final concentration 1000 IU/ml) at 4 °C. Samples were then filtered through a 200-μm cell strainer (43–50,200; pluriSelect) and homogenized in a Dounce homogenizer with the tight head pestle. Samples were incubated on ice for 5 min in 4 ml of EZ Lysis buffer, then filtered through a 40-μm cell strainer (43–50,040; pluriSelect) and centrifuged at 500 g for 5 min at 4 °C. The resuspended pellet was then washed with 4 ml of lysis buffer and incubated for 5 min at 4 °C. The sample was centrifuged again and resuspended in Diluted Nuclei Buffer (PN-2000153; 10X Genomics) and filtered through a 5-μm cell strainer (43–50,005; pluriSelect). After counting, nuclei suspensions were diluted as needed to target 10,000 nuclei per lane and loaded into a thermal cycler to begin the transposition reaction. The manufacturer’s protocol was followed for the completion of library preparation.

For snATAC-seq, samples were chopped into < 2 mm pieces, homogenized with a Dounce homogenizer (885,302–0002; Kimble Chase) in 2 ml of ice-cold Nuclei EZ Lysis buffer (PN-2000207; Sigma-Aldrich) with protease inhibitor (5,892,791,001; Roche) and incubated on ice for 5 min. The homogenate was filtered through a 40-μm cell strainer (43–50,040–51; pluriSelect) and centrifuged at 500 × g for 5 min at 4 °C. The pellet was resuspended, washed with 4 ml of buffer, and incubated on ice for 5 min. Following centrifugation, the pellet was resuspended in Nuclei Buffer (10 × Genomics, PN-2000153). The suspension was then filtered through a 5-μm cell strainer (43–50,005-03, pluriSelect) and counted. After counting, nuclei suspensions were diluted to target 10,000 nuclei per lane and loaded into a thermal cycler to begin the transposition reaction. The manufacturer’s protocol was followed for the completion of library preparation.

### Single nucleus multiome sequencing and bioinformatics workflow

Nine single nucleus multiome libraries were analyzed using 10X Chromium Single Cell Multiome ATAC + Gene Expression v1 chemistry following nuclear dissociation (10X Genomics, Pleasanton, CA). These libraries included 5 male and 4 female samples from control donors and donors with CKD. Three of these libraries were prepared for a prior study and downloaded from the sequence read archive (GSE220289). A target of 10,000 nuclei per lane were loaded onto a thermal cycler for index PCR per manufacturer’s instructions. Libraries were sequenced on an Illumina Novaseq instrument, demultiplexed with bcl2fastq, and counted with cellranger-arc v2.0 (10X Genomics) using the refdata-cellranger-arc-GRCh38-2020-A-2.0.0 reference. Libraries were aggregated with cellranger-arc without depth normalization. A mean of 400,600,460 reads per library were sequenced for the ATAC modality (s.d. = 129,572,369) corresponding to a median of 14,122 fragments per cell (s.d. = 8192). A mean of 441,862,005 reads per library were sequenced for the RNA modality (s.d. = 360,140,888) corresponding to a median of 2792 genes per cell (Additional File 1). Aggregated datasets were processed with Seurat v4.1.0 and its companion package Signac v1.6.0 [64]. A Seurat object was created using the CreateSeuratObject function and preprocessed using the NucleosomeSignal and TSSEnrichment functions. Low-quality nuclei were filtered with the following parameters: nCount_ATAC < 100,000, nCount_RNA < 20,000, nCount_ATAC > 1000, nCount_RNA > 1000, nucleosome_signal < 1, and TSS.enrichment > 2. The RNA assay was normalized using the SCTransform function followed by PCA, and the ATAC assay was normalized using term-frequency inverse-document-frequency (TFIDF) followed by singular value decomposition (SVD) of the TFIDF matrix. Joint integration was performed using the FindIntegrationAnchors function with regularized latent semantic indexing followed by IntegrateEmbeddings. Doublets were identified using AMULET v1.1.0 and DoubletFinder v2.0.3 and removed prior to downstream processing [65, 66]. Harmony v0.1.0 was used for batch correction of the integrated embeddings [67]. Dimensional reduction was performed with the FindNeighbors function using dimensions 2:30 and clustered using the FindClusters function with the Louvain algorithm. UMAP was performed using the harmony reduction and dimensions 2:30. A previously published snRNA-seq atlas of control and CKD donors was used for label transfer and cells were annotated using established lineage-specific markers [23].

In the final object, there was a mean of 6387 ± 4068 nuclei per multiome library with a mean of 6078 ± 3617 peaks and a median of 2517 genes detected per nucleus. The final ATAC assay had a total of 250,009 unique peak regions among 57,491 nuclei and represented all major cell types within the kidney cortex. Representative quality control plots are in Additional File 2: Fig S1. Differential gene expression and chromatin accessibility between cell types was assessed with the FindMarkers function using a Bonferroni-adjusted Wilcoxon Rank Sum test to determine significance at an FDR < 0.05. chromVAR (1.14.0) motif activities were computed using the Signac wrapper and JASPAR2020 database (v0.99.10) adjusted for the number of fragments in peaks for each nucleus [68].

Cell-specific DEG with an adjusted *p*-value less than 0.05 were used as input to the gseGO function in clusterProfiler to identify enriched biological processes [69]. Activated and suppressed biological processes that met the adjusted *p*-value threshold were graphed according to effect size.

### Single nucleus ATAC sequencing and bioinformatics workflow

Twenty-two snATAC-seq libraries were analyzed with 10X Genomics Chromium Single Cell ATAC v1 chemistry following nuclear dissociation. These libraries included twelve control and ten DKD samples. Seventeen of these libraries were prepared for prior studies and downloaded from the sequence read archive (GSE151302, GSE195460, GSE172008, GSE200547) [22, 23, 70, 71]. A target of 10,000 nuclei were loaded onto each lane. Sample index PCR was performed at 12 cycles. Libraries were sequenced on an Illumina Novaseq instrument, demultiplexed with bcl2fastq, and counted with cellranger-atac v2.0 (10X Genomics) using the refdata-cellranger-arc-GRCh38-2020-A-2.0.0 reference. Libraries were aggregated with cellranger-atac without depth normalization. A mean of 396,621,778 reads were sequenced for each snATAC library (s.d. = 150,209,872) corresponding to a median of 18,634 fragments per cell (s.d. = 11,800). Aggregated datasets were processed with Seurat v4.1.0 and its companion package Signac v1.6.0. A Seurat object was created using the CreateSeuratObject function with min.cells = 10 and min.features = 200. Low-quality cells were removed from the aggregated snATAC-seq dataset (nucleosome_signal < 4, TSS.enrichment > 2, pct_reads_in_peaks > 30) before normalization with term-frequency inverse-document-frequency (TFIDF) with default parameters. Dimensional reduction was performed via singular value decomposition (SVD) of the TFIDF matrix. Batch effect was corrected with Harmony v0.1.0 using the lsi reduction. Dimensional reduction was performed with the FindNeighbors function using dimensions 2:30 and clustered using the FindClusters function with the Louvain algorithm. UMAP was performed using the harmony reduction and dimensions 2:30. Homotypic and heterotypic doublets were identified by running AMULET (v1.1.0) on individual snATAC-seq libraries and visualized in the aggregated object prior to removal of doublets with a qval < 0.05. A gene activity matrix was constructed by counting ATAC peaks within the gene body and 2 kb upstream of the transcriptional start site using protein-coding genes annotated in the Ensembl database. The gene activity matrix was log-normalized prior to label transfer with a previously published snRNA-seq Seurat object of control and CKD kidney using canonical correlation analysis [23]. The aggregated snATAC-seq object was filtered using label transfer to remove additional heterotypic doublets not captured by AMULET.

In the final object, there was a mean of 7627 ± 4121 nuclei per snATAC-seq library with a mean of 8098 ± 6905 peaks detected per nucleus. The final snATAC-seq library contained a total of 287,606 unique peak regions among 167,772 nuclei and represented all major cell types within the kidney cortex. Representative quality control plots are in Additional File 2: Fig S5. Differential chromatin accessibility between cell types was assessed with the FindMarkers function using a Bonferroni-adjusted Wilcoxon Rank Sum test to determine significance at an FDR < 0.05. chromVAR (1.14.0) motif activities were computed using the Signac wrapper and JASPAR2020 database (v0.99.10) adjusted for the number of fragments in peaks for each nucleus.

### Single-cell RNA bioinformatics workflow

A preprocessed Seurat object (c798e11b-bbde-45dd-bd91-487f27c93f8f_WashU-UCSD_HuBMAP_KPMP-Biopsy_10X-R_12032021.h5Seurat) was downloaded from the KPMP data repository at the following link: https://atlas.kpmp.org/repository/. We used the Seurat functions NormalizeData, FindVariableFeatures, ScaleData, and RunPCA to prepare the dataset for label transfer. We used a previously published Seurat object of control and CKD donors to transfer cell type annotations with the FindTransferAnchors and TransferData functions [23]. We subsequently filtered the KPMP object using the following parameters: prediction.score.max < 0.5, percent.mt < 10 and nCount_RNA < 10,000 and performed batch correction with Harmony v0.1.0. Clustering was performed by constructing a KNN graph with the Louvain algorithm using dimensions 1:30. Dimensional reduction was performed with the RunUMAP function using dimensions 1:26 and individual clusters were annotated based on the expression of lineage-specific markers.

In the final object, there was a mean of 4421 ± 3449 cells per scRNA-seq library and a mean of 1829 ± 928 genes detected per cell. Representative quality control plots are in Additional File 2: Fig S8. The final snRNA-seq library contained 128,232 cells and represented all major cell types within the kidney cortex. Differential expression between cell types was assessed with the Seurat FindMarkers function using a Wilcoxon Rank Sum test. Bonferroni-adjusted *p*-values were used to determine significance at an FDR < 0.05.

### LOY detection by digital PCR

Genomic DNA was isolated from kidney cortex samples or cell culture lines using a Qiagen DNeasy Blood and Tissue Kit (Qiagen, ID:69,504) and eluted in nuclease free water (Invitrogen, 10–977-015). DNA concentration was measured using a Qubit 4 fluorometer (ThermoFisher, Q33240) according to manufacturer’s instructions. Digital PCR was performed on the Quantstudio Absolute Q dPCR System (ThermoFisher, A52864) using custom Taqman MGB primers and probes targeting chrY and chrX. The FAM-labeled chrY probe targeted the first exon of *SRY* on the long arm of the Y chromosome: chrY-FWD-TGTGCCTCCTGGAAGAATGG, chrY-REV-GATCAGCAAGCAGCTGGGATA, chrY-PROBE-CATTTTTCGGCTTCAGTAAG. The ABY-labeled chrX probe targeted a region near *TSIX* on the long arm of the X chromosome: chrX-FWD-TCAAGAGGGATGGACAAAGGA, chrX-REV-AAAGCAGGTGAGGCGGTAAG, chrX-PROBE-CAGAAGACACTCAAGAAT. Each dPCR reaction had 150 ng DNA diluted in 7 µl of nuclease free water, 0.5 µl of 20X FAM-labeled chrY primer and probe mix, 0.5 µl of 20X ABY-labeled chrX primer and probe mix, 2 µl of 5X DNA master mix (ThermoFisher, A52490), and 15 µl of Absolute Q Isolation Buffer (ThermoFisher, A52730). Reactions were loaded into a QuantStudio Absolute Q MAP16 Plate (ThermoFisher, A53301). The reaction was preheated to 96 °C for 10 min, and PCR was run for 40 cycles alternating between (1) 96 °C for 5 s and (2) 60 °C for 15 s. Data was analyzed using the Quantstudio software and visualized in R.

### Spatial Visium sample preparation and bioinformatics workflow

Eight spatial libraries were prepared with 10X Genomics Visium v1.0 chemistry. Paraffin-embedded kidney samples were cut into 5 μm-thick sections and mounted onto the active sequencing areas (6 mm × 6 mm) of the 10 × Genomics Visium slides. All steps for tissue adherence, hematoxylin and eosin staining, imaging and probe hybridization, elongation, and recovery were per 10 × Visium v1.0 protocol. Libraries were generated using sample-specific TS dual index 10-base UMI and amplified by sample-specific cycle numbers. Quality control was confirmed by Agilent Tapestation High Sensitivity D5000 analysis (1:5 dilution). Spatial sequencing libraries were sequenced in the Washington University sequencing core on a NovaSeq S4 instrument according to manufacturer’s instructions using dual indexing. Libraries were demultiplexed with bcl2fastq and counted with spaceranger v2.0 (10X Genomics) using the refdata-gex-GRCh38-2020-A reference. Individual libraries were preprocessed using Seurat with the NormalizeData, ScaleData, FindVariableFeatures, and RunPCA functions. Libraries were integrated using the FindIntegrationAnchors and IntegrateData functions. Harmony v0.1.0 was used for batch correction of the integrated PCA and clustered using the FindNeighbors, FindClusters, and RunUMAP functions. We used the processed KPMP dataset to transfer cell type annotations to spatial spots using the FindTransferAnchors and TransferData functions. We annotated each of the spots using lineage-specific markers that correspond to cell types from the single-cell datasets.

In the final object, there was a mean of 2701 ± 755 spots per spatial library and a mean of 3192 ± 2561 genes detected per spot. Representative quality control plots are in Additional File 2: Fig S10. The final spatial library contained 21,611 spots and represented all major cell types within the kidney cortex. Differential expression between spot neighborhoods was assessed with the Seurat FindMarkers function. Bonferroni-adjusted *p*-values were used to determine significance at an FDR < 0.05.

We used CellChat to quantify intercellular ligand-receptor interactions between neighboring spots [35]. CellChat was run on spaceranger output for individual spatial libraries using the secreted signaling database. CellChat objects were created with the createCellChat function. Spot interaction probabilities were calculated with the computeCommunProb function with the following parameters recommended for Visium datasets: type = “truncatedMean”, trim = 0.1, distance.use = TRUE, interaction.length = 200, scale.distance = 0.01. Cell communication networks were aggregated with the aggregateNet function and network centrality scores were computed. CellChat objects were merged with the mergeCellChat function and the interaction probability between spot neighborhoods was computed for each secreted signaling network and Visium dataset.

We used the Seurat FindMarkers function to identify neighborhood-specific differentially expressed genes (DEG) using a Bonferroni-adjusted Wilcoxon Rank Sum test. Neighborhood-specific DEG with an adjusted *p*-value less than 0.05 were used as input to the gseGO function in clusterProfiler to identify enriched biological processes. Activated and suppressed biological processes that met the adjusted *p*-value threshold were graphed according to effect size.

### Statistical models for single-cell LOY detection

For snATAC-seq libraries and the ATAC modality of single-cell multiomes, we quantified ATAC fragments in 1 Mb bins after excluding highly repetitive regions [42]. Cells with at least 10,000 fragments mapping to these regions were included for downstream analysis. The number of fragments mapping to each chromosome was normalized by the total number of fragments per cell and log-transformed after adding a pseudocount of 1. Datasets were aggregated and the median normalized chromosome coverage was computed for each cell type to calculate a ratio between the global and cell-specific median. This ratio was used to correct chromosome coverage across cell types and adjust for cell-specific chromatin accessibility. The corrected coverage was scaled for each chromosome and donor and used to estimate a kernel density in R. For unimodal LOY determination, the trough of the kernel density was used as a threshold.

For scRNA-seq libraries and the RNA modality of single-cell multiomes, we quantified the number of Y chromosome transcripts, divided by the total number of transcripts, and log-transformed the result after adding a pseudocount of 1. Datasets were aggregated and the median normalized chromosome coverage was computed for each cell type to calculate a ratio between the global and cell-specific median. This ratio was used to correct chromosome coverage across cell types and adjust for cell-specific Y chromosome expression. The corrected coverage was scaled for each chromosome and donor and used to estimate a kernel density in R. For unimodal LOY determination, the trough of the kernel density was used as a threshold.

For single-cell multiomes, the scaled Y chromosome ATAC fragments and RNA counts were used for a joint LOY estimate. We used mclust to implement a two-state semi-supervised gaussian mixture model with spherical varying volume [72]. Cells at the origin (LOY, i.e., no coverage for either modality) or cells with greater than median coverage for either the ATAC or RNA modality (XY) were assigned to one of two initial states. The model was used to classify male cells as XY or LOY and these genotypes were used for downstream analyses.

### Single-cell autosomal CNV detection

For snATAC-seq libraries and the ATAC modality of single-cell multiomes, epiAneufinder was used to call autosomal CNV in 1 Mb bins after excluding highly repetitive regions [42]. Cells with at least 10,000 fragments mapping to these regions were included for downstream analysis, excluding sex and mitochondrial chromosomes. Libraries were analyzed individually and autosomal CNV were called by comparing the coverage in each 1 Mb bin to a population mean after segmentation and GC correction. Single-cell CNV burden was the proportion of 1 Mb bins that had either a gain or loss in each cell.

### GLMM to identify variables associated with LOY

We used a generalized linear model with a logit link function and mixed effect per donor to determine if cell type, age, and CNV burden were significantly associated with LOY [73]. Model parameters and equations are summarized in Additional File 12. Odds ratios and 95% CI were used to determine significance of predictor variables. Estimated marginal means were obtained from the plot_model function in sjPlot.

### Supplementary Information

**Additional file 1. **Single-cell and spatial library metadata and demographics (Table S1). Library quality control metrics (Tables S2-S4) and cell type distribution (Tables S5-S8).

**Additional file 2. **Supplemental figures.

**Additional file 3. **Single-cell multiome cell-specific differentially expressed genes (DEG) for LOY vs XY.

**Additional file 4. **Single-cell multiome age-adjusted cell-specific differentially expressed genes (DEG) for LOY vs XY.

**Additional file 5. **Single-cell multiome cell-specific differentially accessible regions (DAR) for LOY vs XY.

**Additional file 6. **Single-cell multiome cell-specific chromVAR differential motif activity for LOY vs XY.

**Additional file 7. **snATAC-seq cell-specific differentially accessible regions (DAR) for LOY vs XY.

**Additional file 8. **snATAC-seq cell-specific chromVAR differential motif activity for LOY vs XY.

**Additional file 9. **KPMP scRNA-seq cell-specific differentially expressed genes (DEG) for LOY vs XY.

**Additional file 10. **KPMP scRNA-seq age-adjusted cell-specific differentially expressed genes (DEG) for LOY vs XY.

**Additional file 11. **Joint multiome and KPMP sc/snRNA-seq age-adjusted cell-specific differentially expressed genes (DEG) for LOY vs XY.

**Additional file 12. **Generalized linear mixed model results for LOY.

**Additional file 13. **Review history.

## Data Availability

All of the data for this manuscript have been made publicly available (see Additional File 1 for a table outlining data types and location). Raw sequencing data for single-cell multiomes and snATAC-seq are deposited in GEO under accession number GSE232222 [76]. Raw sequencing and processed data for spatial Visium libraries are deposited under accession number GSE232431 [77]. Previously published raw sequencing data for single-cell multiomes and snATAC-seq are available in GSE220289, GSE151302, GSE195460, GSE172008, and GSE200547 [78–82]. All other relevant data supporting the key findings of this study are available within the article and its Supplementary Information files.
