# Supplementary material for: Mosaic loss of Y chromosome is associated with aging and epithelial injury in chronic kidney disease
Source: Genome Biol. 2024 Jan 29;25:36. doi: 10.1186/s13059-024-03173-2 (PMC10823641; doi:10.1186/s13059-024-03173-2)
Supplement: Supplementary file 2 — Additional file 2. Supplemental figures. [file 13059_2024_3173_MOESM2_ESM.docx]

**Mosaic loss of Y chromosome is associated with aging and epithelial injury in chronic kidney disease**

Parker C. Wilson, Amit Verma, Yasuhiro Yoshimura, Yoshiharu Muto, Haikuo Li, Nicole P. Malvin, Eryn E. Dixon and Benjamin D. Humphreys

**Supplemental Figures**


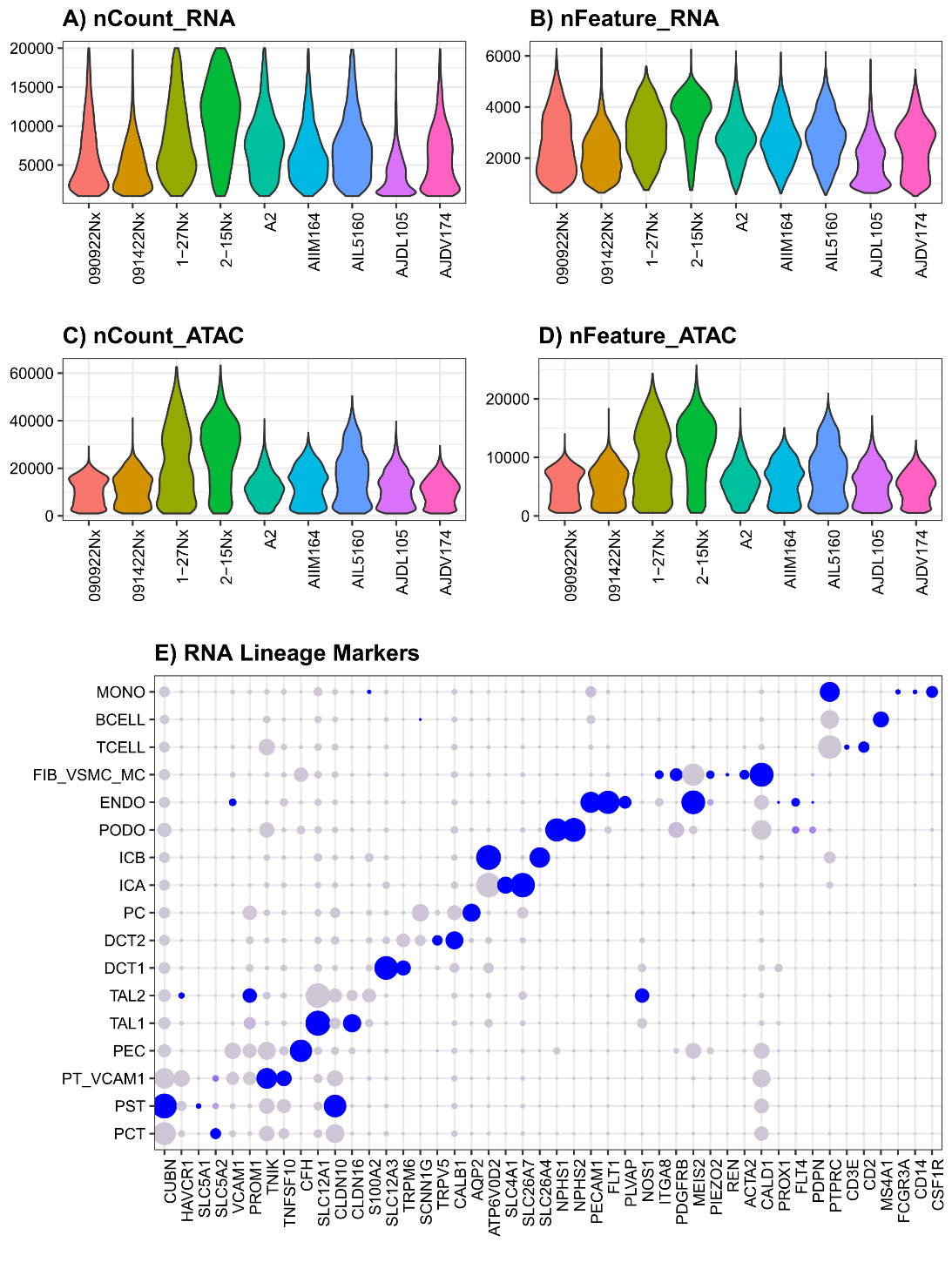


**Fig S1 – Single nucleus multiome quality control metrics A)** Number of RNA transcripts per nucleus by donor **B)** Number of genes detected per nucleus by donor **C)** Number of ATAC fragments per nucleus by donor **D)** Number of peaks detected per nucleus by donor **E)** Lineage-specific marker expression in the RNA assay. MONO – mononuclear leukocytes, BCELL – B cells, TCELL – T cells, FIB_VSMC_MC – fibroblasts, vascular smooth muscle and mesangial cells, ENDO – endothelium, PODO – podocytes, ICA – type A intercalated cells, ICB – type B intercalated cells, PC – principal cells, DCT2 –late distal convoluted tubule, DCT1 – early distal convoluted tubule, TAL1,2 –thick ascending limb, ATL – ascending thin limb, PEC – parietal epithelium, PT_VCAM1 – VCAM1+ proximal tubule, PST – proximal straight tubule, PCT – proximal convoluted tubule


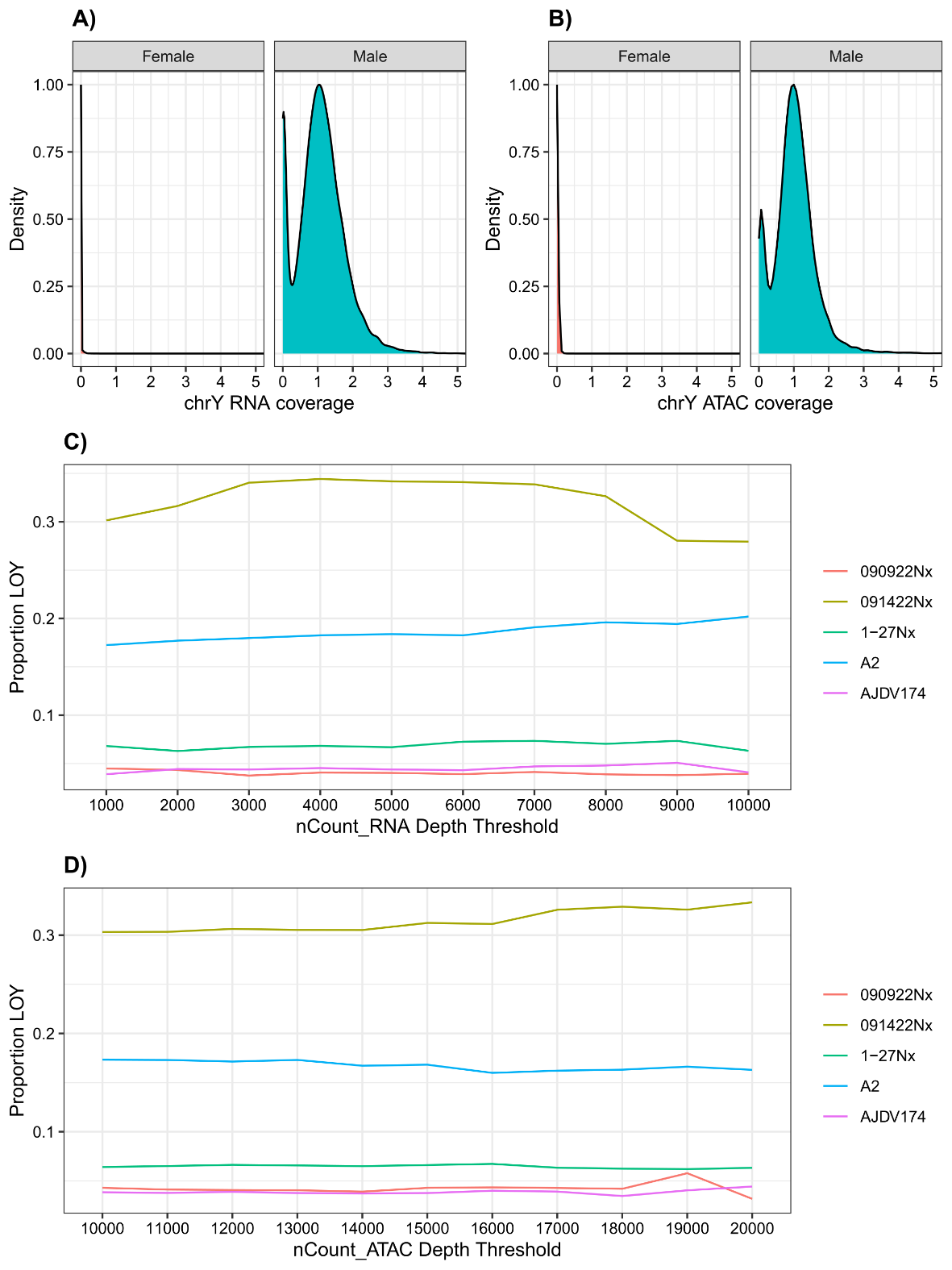


**Fig S2 – LOY detection by single cell multiome sequencing A)** Y chromosome RNA transcript density normalized by single nucleus sequencing depth and scaled by donor **B)** Y chromosome ATAC fragment density normalized by single nucleus sequencing depth and scaled by donor **C)** Proportion LOY in proximal tubule in male donors by sequencing depth threshold for RNA assay **D)** Proportion LOY in proximal tubule in male donors by sequencing depth threshold for ATAC assay


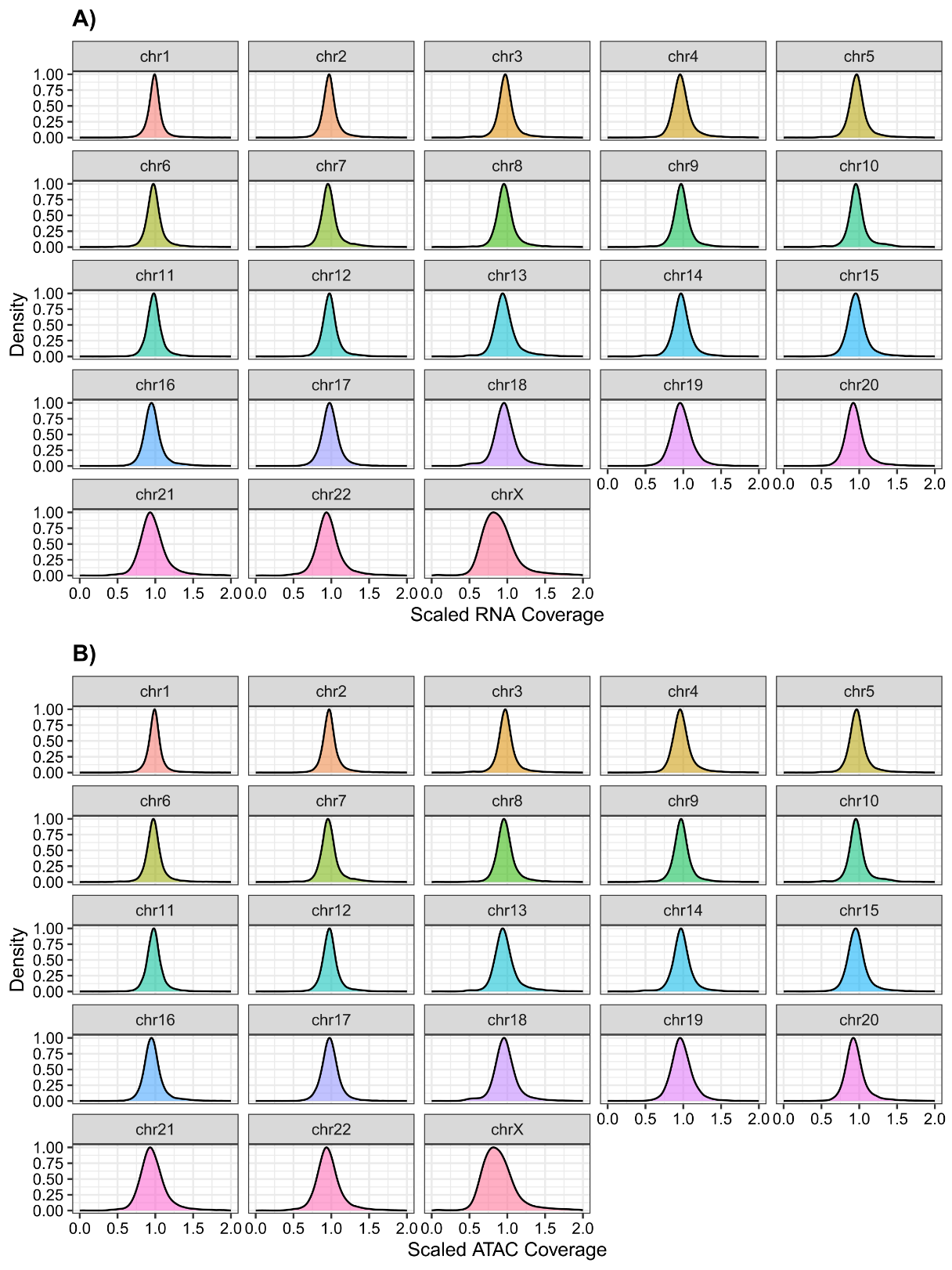


**Fig S3 – Single cell multiome chromosome density plots A)** RNA assay **B)** ATAC assay


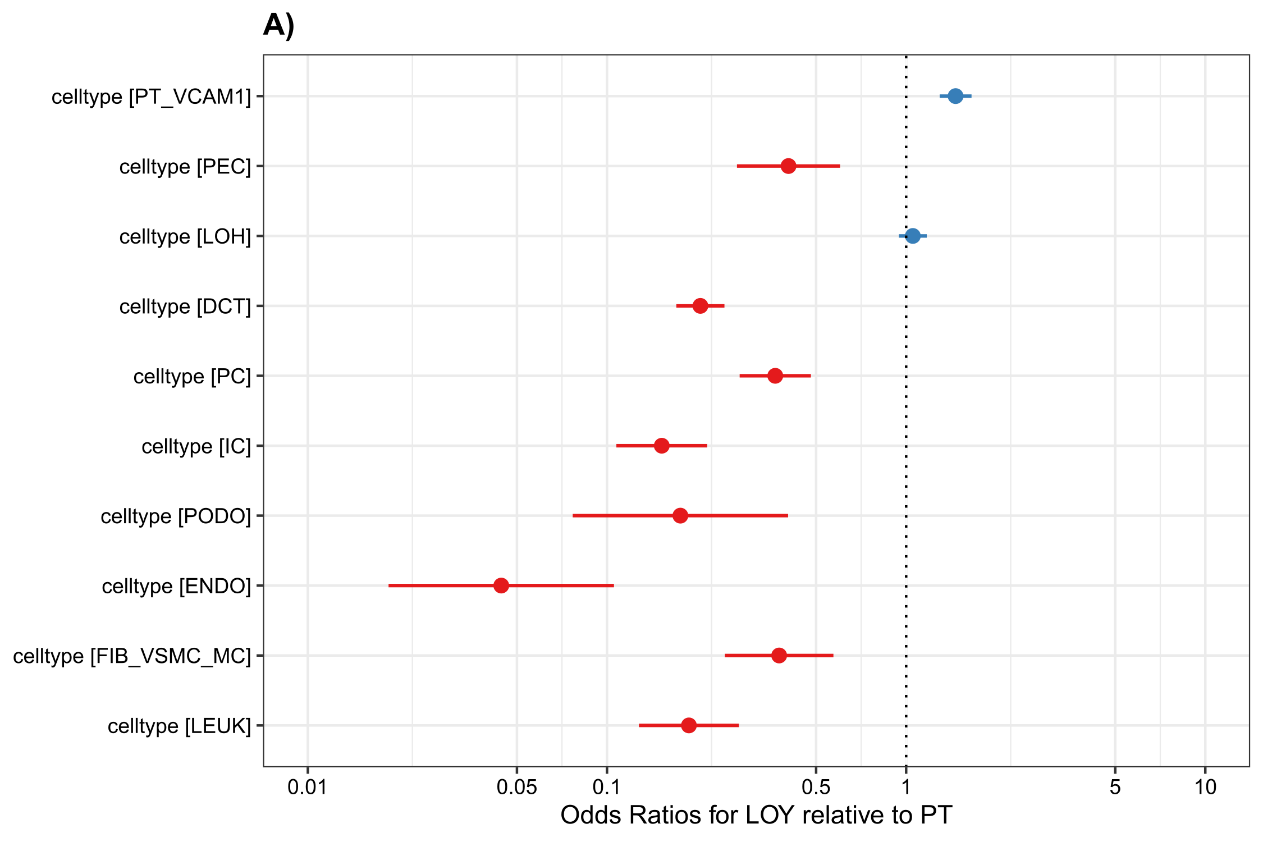


**Fig S4 – Generalized linear mixed model for LOY in single cell multiomes A)** GLMM by cell type relative to the proximal tubule (PT). Odds ratios are displayed with 95% CI.


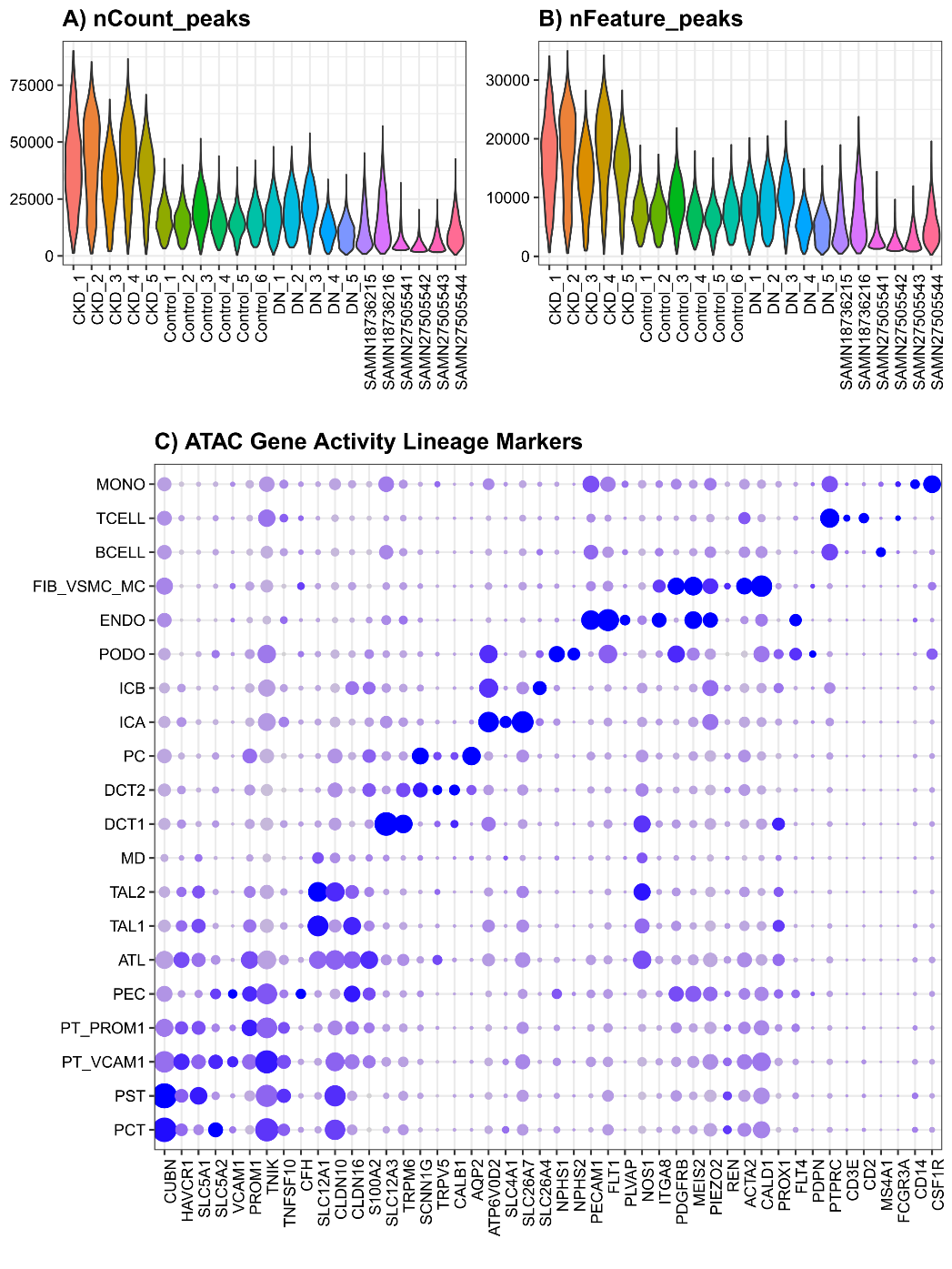


**Fig S5 – Single nucleus ATAC quality control metrics A)** Number of ATAC peaks per nucleus by donor **B)** Number of ATAC fragments detected per nucleus by donor **C)** Lineage-specific gene activity in the ATAC assay. MONO – mononuclear leukocytes, TCELL – T cells, BCELL – B cells, FIB_VSMC_MC – fibroblasts, vascular smooth muscle and mesangial cells, ENDO – endothelium, PODO – podocytes, ICA – type A intercalated cells, ICB – type B intercalated cells, PC – principal cells, DCT2 –late distal convoluted tubule, DCT1 – early distal convoluted tubule, TAL1,2 –thick ascending limb, ATL – ascending thin limb, PEC – parietal epithelium, PT_PROM1 – PROM1+ proximal tubule, PT_VCAM1 – VCAM1+ proximal tubule, PST – proximal straight tubule, PCT – proximal convoluted tubule


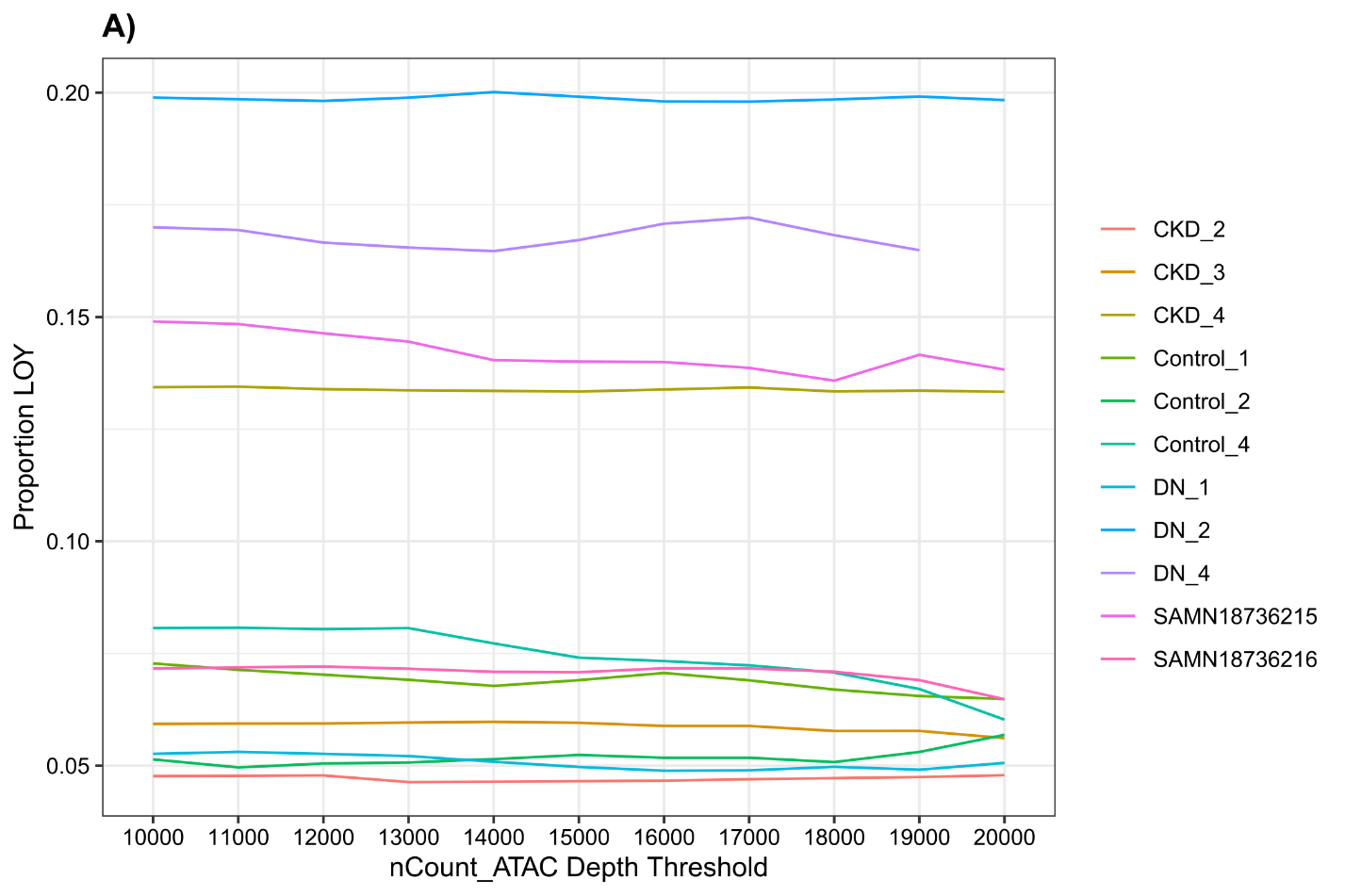


**Fig S6 – LOY detection by single cell ATAC sequencing A)** Proportion LOY in proximal tubule in male donors by sequencing depth threshold for ATAC assay


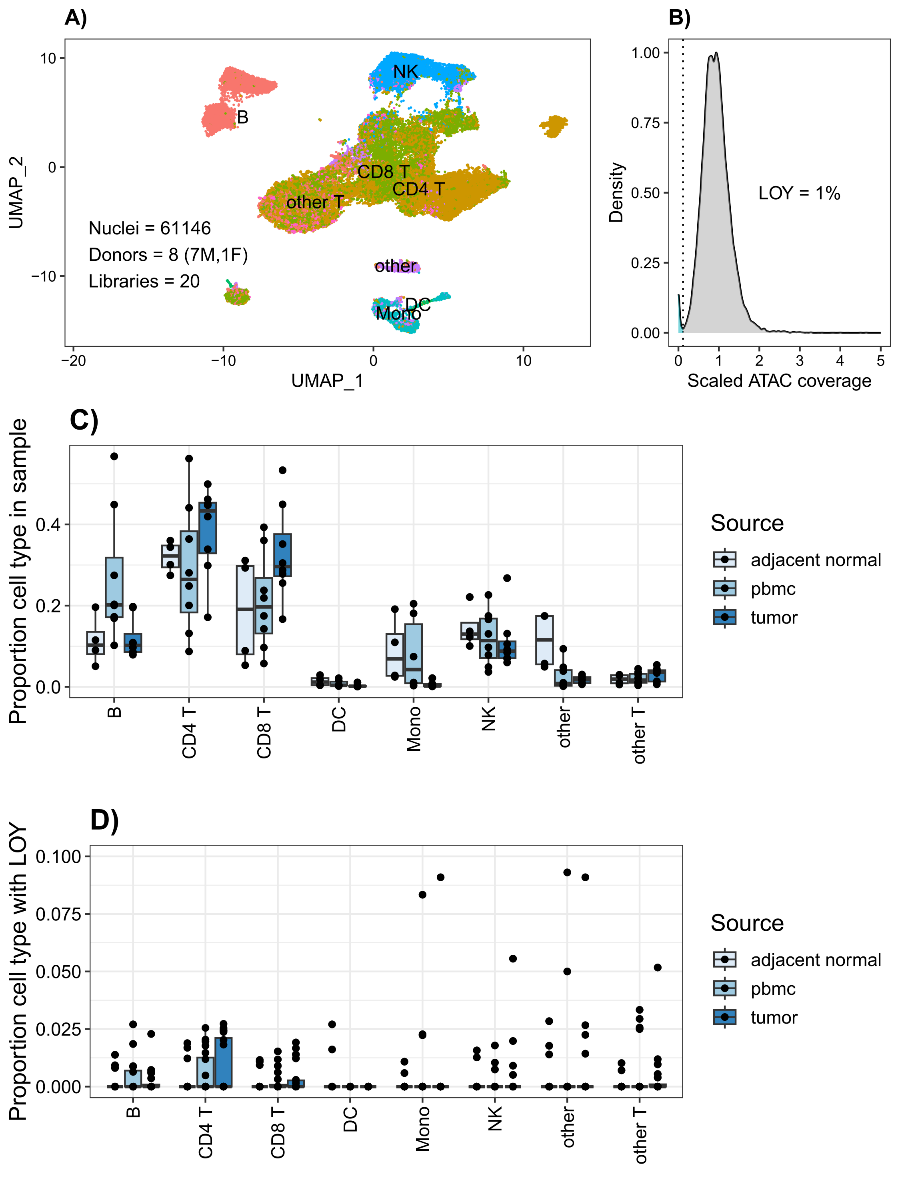


**Fig S7 – LOY detection in CD45+ leukocytes by snATAC-seq A)** UMAP of male and female leukocytes B – B cells, CD8 T – CD8+ T cells, CD4 T – CD4+ T cells, NK – NK cells, other T – T cells, Mono – mononuclear cells, DC – dendritic cells, other – other leukocytes **B)** ATAC modality LOY density plot in 51,898 male cells **C)** Proportion of each leukocyte type by tissue source **D)** Proportion LOY by leukocyte type and tissue source in male cells


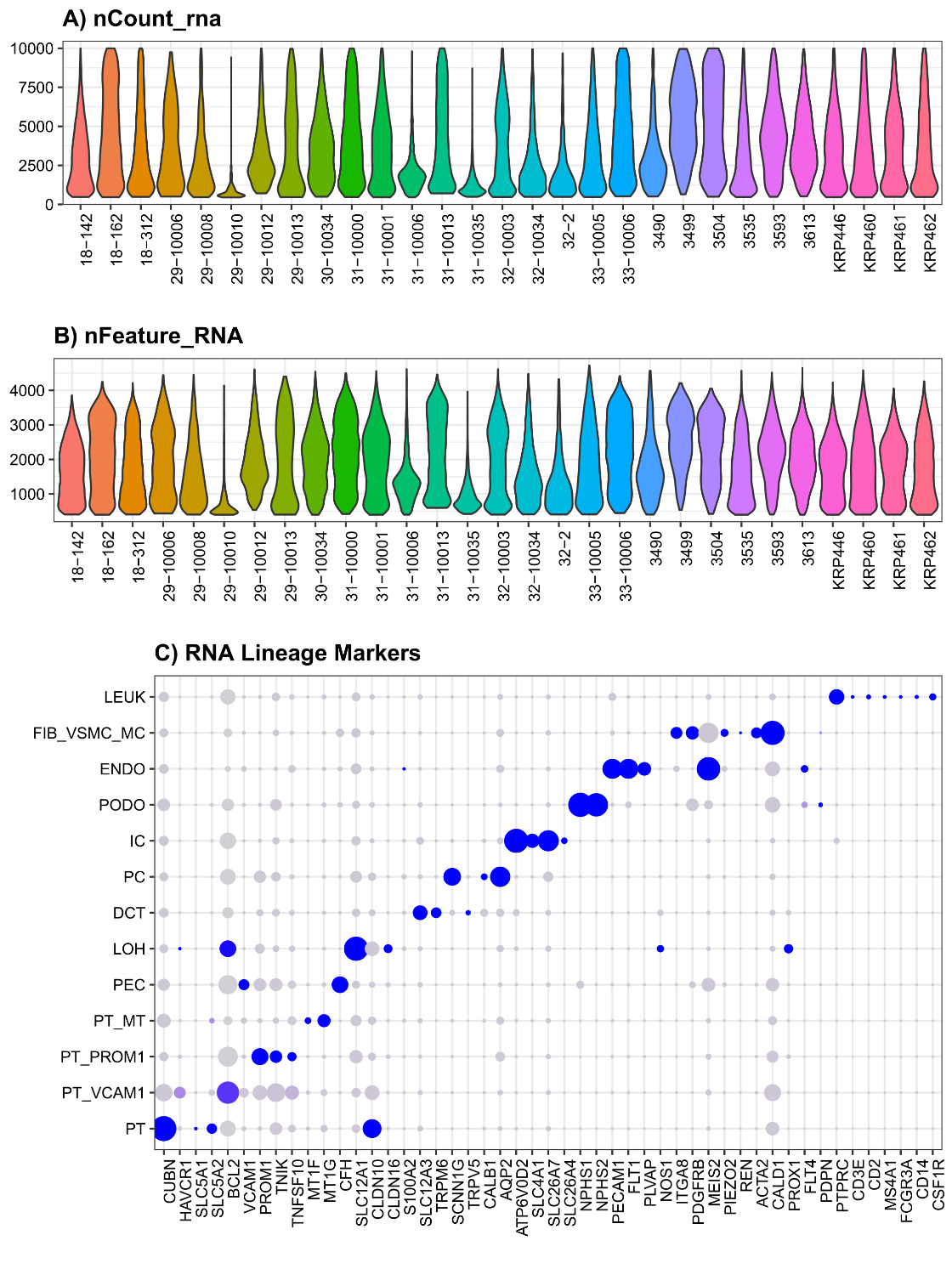


**Fig S8 – KPMP Single cell RNA quality control metrics A)** Number of RNA transcripts per cell by donor **B)** Number of genes detected per cell by donor **C)** Lineage-specific marker expression in the RNA assay, LEUK – leukocytes, FIB_VSMC_MC – fibroblasts, vascular smooth muscle and mesangial cells, ENDO – endothelium, PODO – podocytes, IC – intercalated cells, PC – principal cells, DCT – distal convoluted tubule, LOH – loop of Henle, PEC – parietal epithelium, PT_MT – mitochondria gene proximal tubule, PT_PROM1 – PROM1+ proximal tubule, PT_VCAM1 – VCAM1+ proximal tubule, PT – proximal tubule


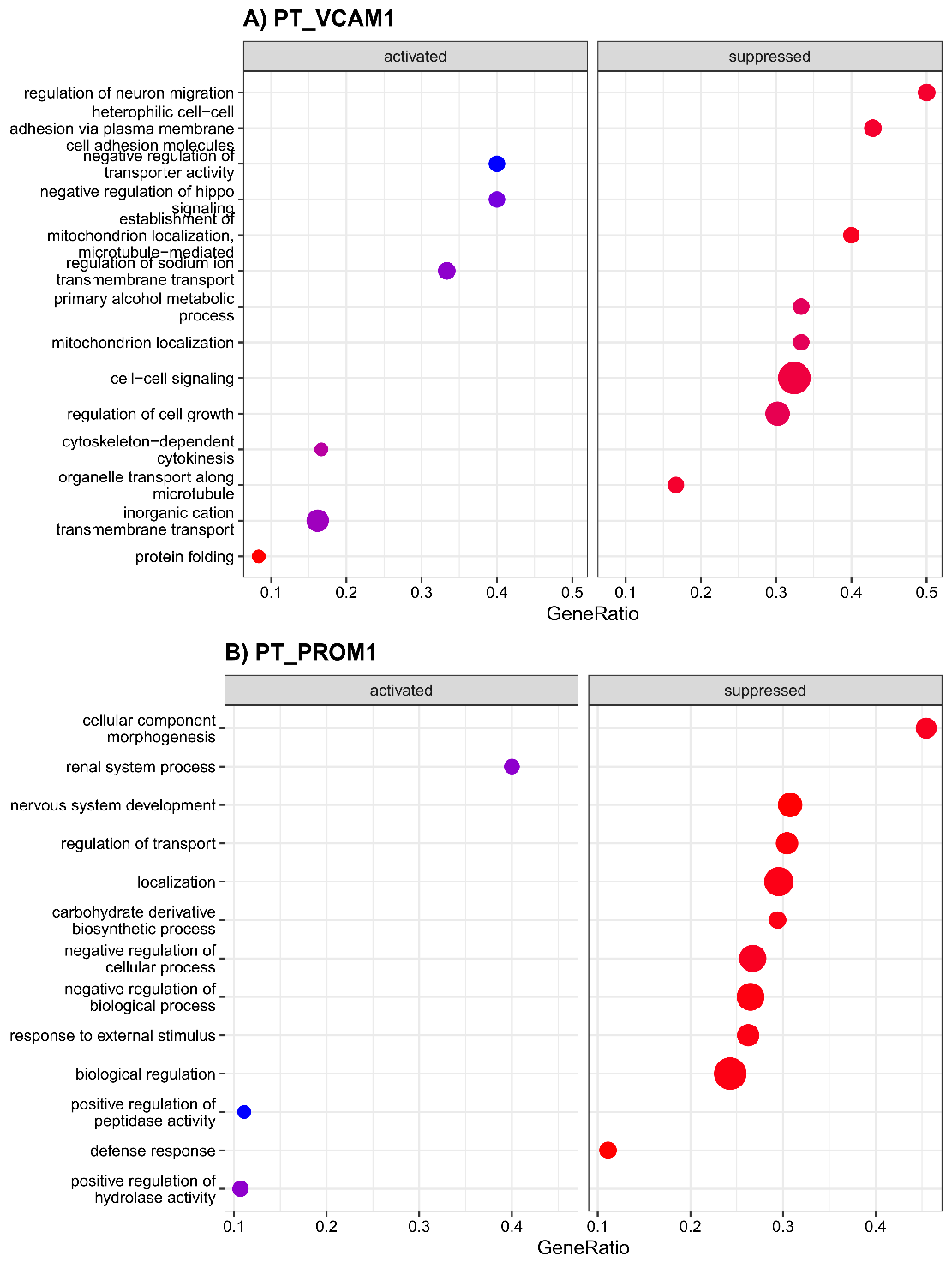


**Fig S9 - GSEA for LOY vs XY using age-adjusted differentially expressed genes in proximal tubule subsets A)** PT_VCAM1 **B)** PT_PROM1


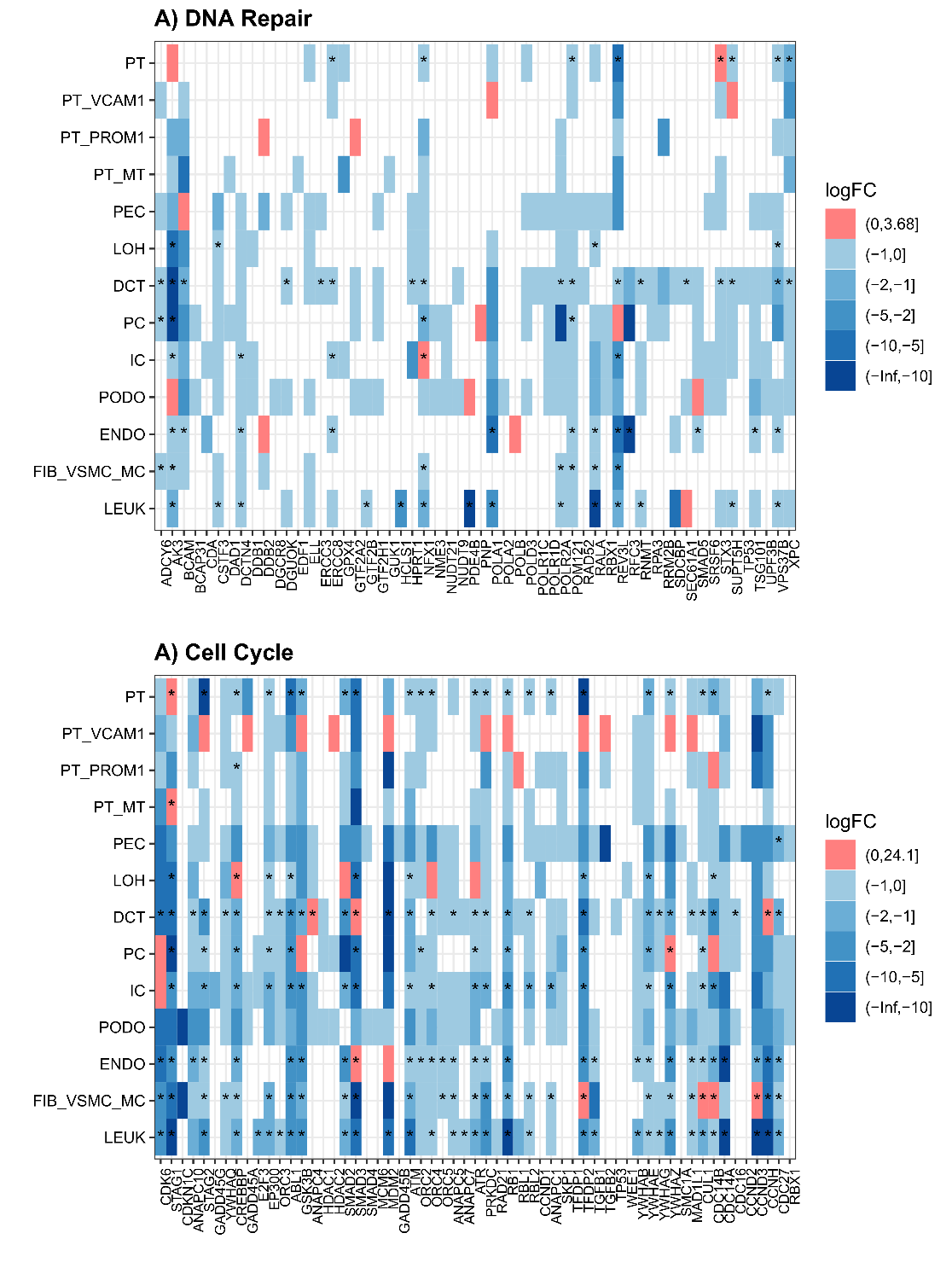


**Fig S10 - Age-adjusted differentially expressed genes for LOY vs XY A)** DNA repair genes **B)** Cell cycle genes. * indicates Bonferroni-adjusted p-value is < 0.05.


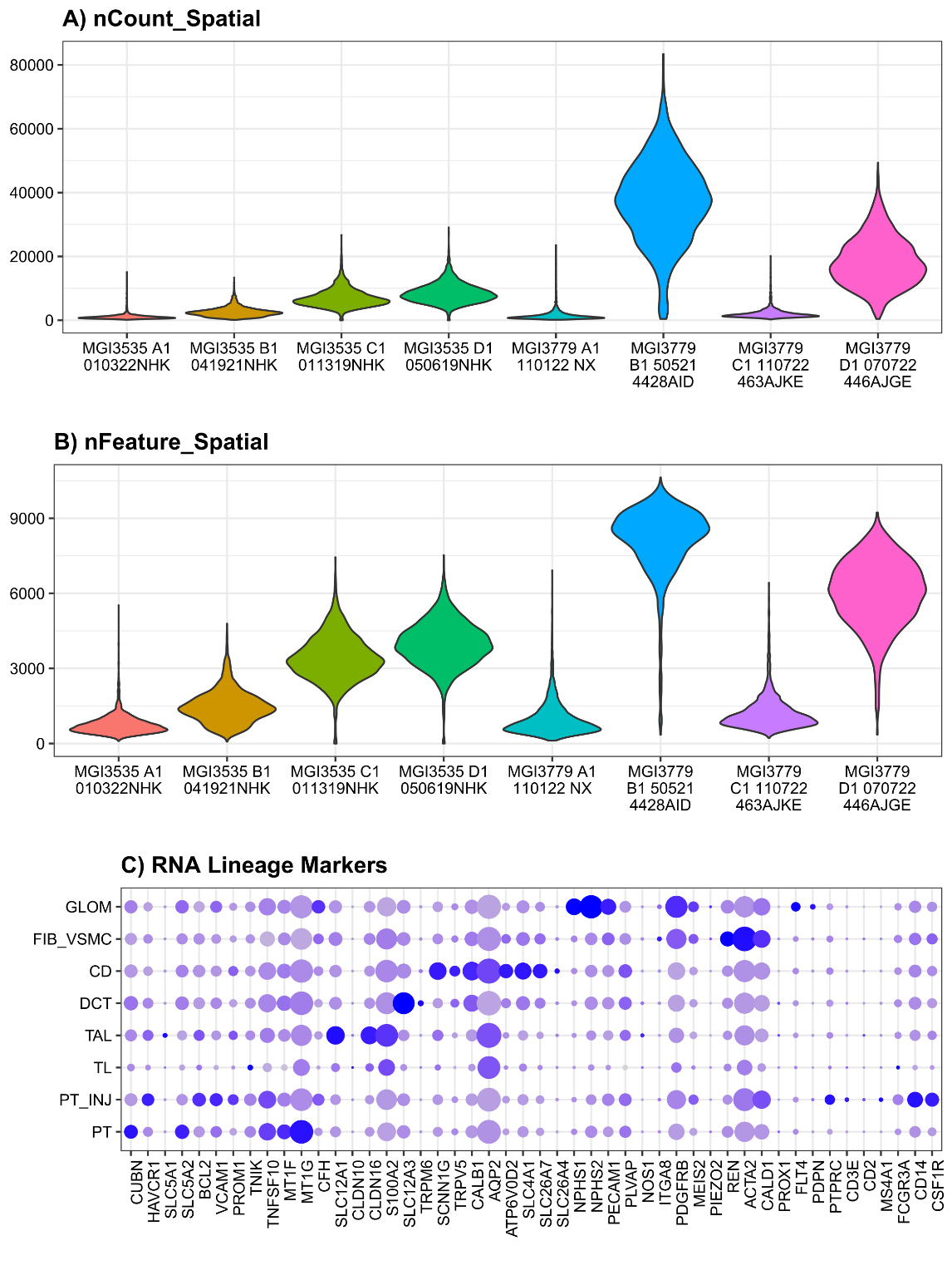


**Fig S11 – Visium Spatial quality control metrics A)** Number of RNA transcripts per spot by donor **B)** Number of genes detected per spot by donor **C)** Lineage-specific marker expression for spot neighborhoods GLOM – glomeruli, FIB_VSMC – fibroblasts and vascular smooth muscle, CD – collecting duct, DCT – distal convoluted tubule, TAL – thick ascending limb, TL – thin limb, PT_INJ – injured proximal tubule, PT – proximal tubule


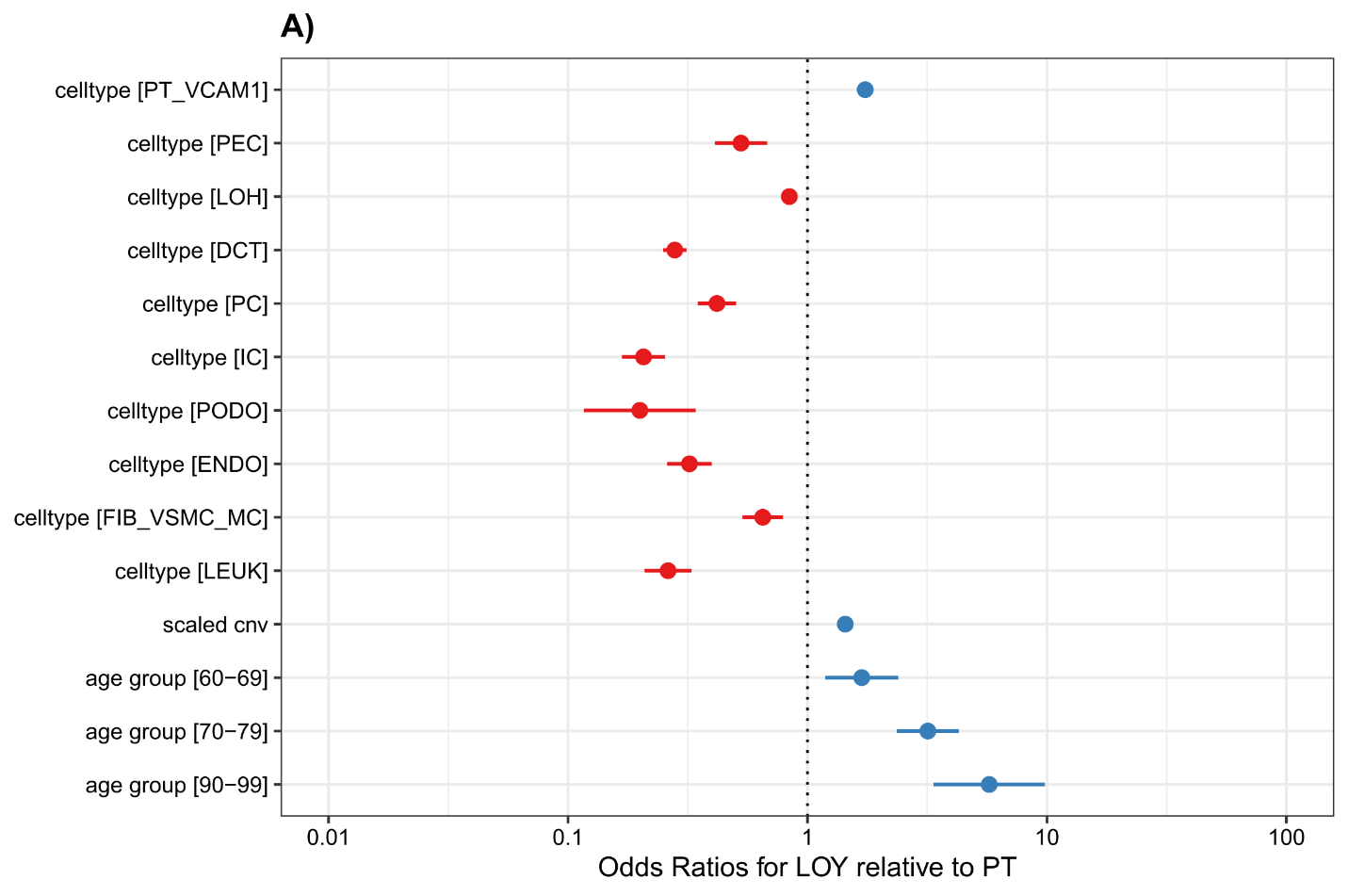


**Fig S12 – Generalized linear mixed model for LOY A)** GLMM by cell type relative to the proximal tubule (PT) adjusted for donor age and scaled CNV burden in single cell multiome and snATAC-seq datasets. Odds ratios are displayed with 95% CI.


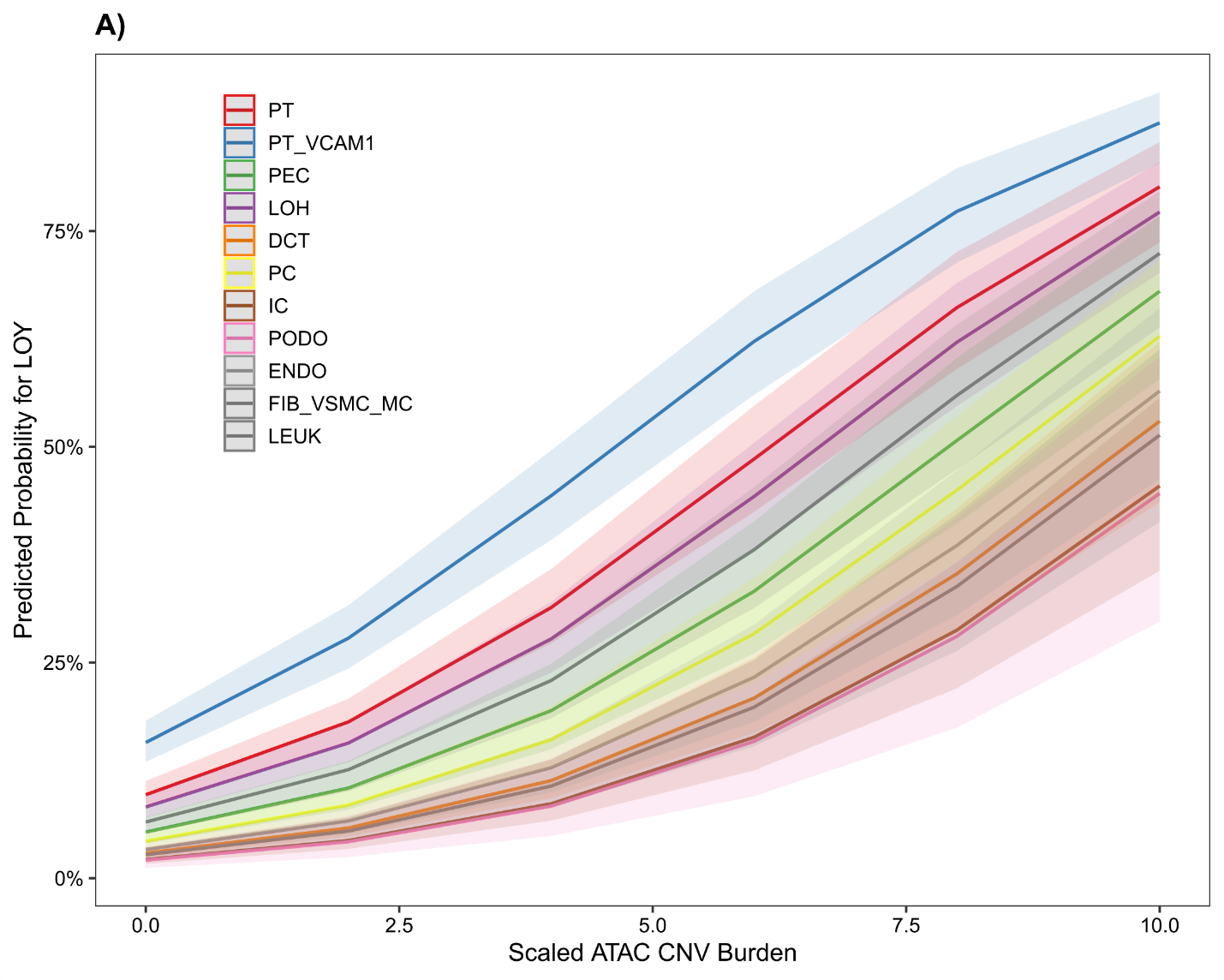


**Fig S13 – Predicted probabilities for LOY by cell type A)** Estimated marginal probabilities for LOY in GLMM for single cell multiome and snATAC-seq datasets adjusted for age, cell type and CNV burden with 95% CI.
