# Supplementary material for: Mosaic loss of Y chromosome is associated with aging and epithelial injury in chronic kidney disease
Source: Genome Biol. 2024 Jan 29;25:36. doi: 10.1186/s13059-024-03173-2 (PMC10823641; doi:10.1186/s13059-024-03173-2)
Supplement: Supplementary file 13 — Additional file 13. Review history. [file 13059_2024_3173_MOESM13_ESM.docx]

**1^st^ round**

**Reviewer 1**

In the manuscript entitled, "Mosaic loss of Y chromosome is associated with aging and epithelial injury in chronic kidney disease" Wilson et al. demonstrate a connection between Y chromosome loss, kidney cell maladaptive repair, and CKD. Although the manuscript is largely technical in nature, demonstrating various transcriptomics techniques can measure LOY, the findings are of interest and have the potential to have a significant impact on the field. The study is well designed, and the data are convincing. That said, for these techniques to show their true value, they need to be compared to the gold standard. Karyotype analysis to ensure the core findings, deletion of the Y chromosome, are reproducible would greatly strengthen the study. These and other concerns are listed below:


1. In the introduction the Authors state, "we describe an approach to detect LOY and other mCA by single cell sequencing." Although the findings are interesting and of potential significance, the scRNA seq technique is not without limitations. If this is to be a novel technique, it should be compared to the gold standard of monitoring LOY, karyotyping, to validate the findings. This doesn't have to be a large number of samples, just enough to confirm LOY is significantly more common in CKD.

2. Is the Y chromosome truly 'lost' or just silenced? For instance, in Figure 3D it is shown LOY is associated with cell division. Is it known whether the Y chromosome is silenced in cell division? This could be addressed in the data from #1 above.

3. There is very little discussion of the limitations of this study. At least some of the data was based on a small number of patients, i.e. n=4 or 5, which could be a limitation. Also, as the paper demonstrates the utility of transcriptomics in analyzing LOY, limitations to the various transcriptomics approaches used should be discussed.

4. It would be interesting to compare the gene ontology pathway enrichment data in 1F and DEG analysis in 3D to cells from female patients, as these cells never had an Y chromosome.

5. Why is LOY worse than never having an Y chromosome in the first place? The data in 3A and SF7 seem to indicate both males and females have PT_VCAM1, PT_PROM1 and PT_MT cell populations at relatively similar rates, but in males, these cells are more likely to have LOY. The Authors should discuss what it is about the LOY that is different than having XX chromosomes.

6. The data in Figure 4 doesn't seem to add much to the current study. There is no mention if the samples are from males or females and there is no connection to the Y chromosome. If staining for the Y chromosome was done prior to the RNA analysis, it could have perhaps strengthened the data.

7. Although there is a clear connection between LOY and kidney failed repair phenotype, it is not clear which comes first. The fact that the majority of the PT_VCAM1 group retains the Y chromosome suggests the loss is occurring after the cells reach the maladaptive repair stage. Also, PT_VCAM1 cells are only approximately twice as likely to have LOY compared to PT group. That said, it is unclear why this small population of maladaptively repaired cells contributes to kidney functional decline in the first place. It is possible that the LOY worsens the failed repair phenotype in this subpopulation. It would be interesting further analyze the DEG to determine if there is a change in failed repair vs failed repair + LOY. In other words, a PT_VCAM1-LOY vs PT_VCAM1-XY (or PT_PROM1-LOY vs PT_PROM1-XY) analysis.


Minor:

1. The "Proportion LOY" data, 1E, 2C, 2C, and etc, isn't clear. Does each dot in a given condition represent a different patient? If so, the numbers don't match the stated n values.

2. It is somewhat confusing to understand which data sets contain data from men and women and which ones are men only. For instance, in the results description for Figure 2 it is stated, "we analyzed 167,772 nuclei from 22 donors, including both men (n=12) and women (n=10)." But a few lines later, "We evaluated male cells for LOY." I take from this only 2A has male and female data, but it is not completely clear. Specifying male vs female or including the n values in the figure legends could make this easier to follow.

**Reviewer 2**

This study examines loss of the Y chromosome (LOY) on single cell RNA and ATAC sequencing in the kidney. The authors speculate that LOY is associated with many phenotypes/outcomes, but no direct measurements are made. For example, claims are made that LOY is an indicator of DNA damage, senescence and disease status, but independent markers for DNA damage and senescence are not measured and a comparison between normal and disease kidney is not made. Specific comments are below:

The association between age and LOY is widely recognized. The current analysis with the kidney dataset was performed at the end of this manuscript and apparently not incorporated into previous analyses of differential gene expression. Thus, it appears that the data does not adjust for age when the association of LOY with changes in gene expression are made. Hence, age can be a confounding factor in the outcomes that are attributed to LOY. In other words: Are the identified pathways caused by LOY? Or are they pathways that increase with age, which is then over-represented in the LOY populations?

The connection between LOY and proliferation in various cell types is based on anecdotal evidence of proliferation. Proliferation is not directly (or indirectly) assessed. For example, were proliferation/cell cycle genes differentially expressed in LOY-positive vs. LOY-negative cells within a particular cell type? This would at least provide indirect support for this claim - that is currently unsubstantiated.

Results presented here suggest that LOY in proximal tubule cells is associated with large transcriptional changes related to cell damage, but the overall conclusion put forth is that LOY is a marker for damaged cells. Could LOY be directly causing these transcriptional changes? Within the PT_VCAM1 population (indicative of damaged cells), what are the differentially expressed genes and transcription factor activities? At a single cell level can you compare DNA damage-associated gene expression in LOY-negative vs. LOY-positive cells?

Studies here are focused on proximal tubule cells, but many other cell types are present in the kidney and could have connections to LOY. In particular, leukocytes have a well-documented LOY burden, and epidemiological studies have provided associations between LOY in blood and many diseases. Except with cancer, these epidemiological associations of LOY with disease do not exist with respect to non-leukocyte cell types, emphasizing the importance of kidney leukocyte analyses.

The spatial transcriptomics analysis does not have the resolution to determine LOY cells (as pointed out in the results).

Sample sizes with cells and patients include men and women, but of course cells from women were not analyzed in downstream analyses on LOY. Thus, it seems that reported sample sizes are misleading.

Transcription factor activity based on open chromatin and transcription factor expression based on RNA profile appear to be confused and are used interchangeably throughout the results.

These authors use their own generated dataset and supplement with other publicly available datasets, but no attempt was made to harmonize the data and combine datasets (that would promote robustness of some conclusions).

**Reviewer 3**

I reviewed the article by Wilson et al. The authors delve into several pertinent questions:
1. The association of LOY with kidney disease.
2. The relationship of LOY to the aging kidney.
3. The cell type where LOY is most prevalent.
4. The chromatin accessibility profile in PT cells with LOY.
5. The transcriptome of PT cells with LOY.
6. The transcriptome of spatial neighborhoods in and around the PT.

While the paper adopts a primarily descriptive approach, this is by no means a limitation. Descriptive studies are foundational in science, often paving the way for hypothesis-driven research. The authors convincingly demonstrate an association between LOY and the aging kidney. It's particularly noteworthy that proximal tubular cells from older kidneys exhibit a higher proportion of LOY. Additionally, adjusted for age, the proximal tubule was identified as the cell type with the highest percentage of cells with LOY.

While not directly studied here, it would be insightful to understand if LOY has been studied in the context of kidney transplantation. Factors such as immune processes, immune reperfusion injury, drug toxicity, and DGF (particularly ATN) might influence the prevalence of LOY ?. This is only pertinent for patients receiving kidneys from male donors. Given that your group possesses data on transplant cohorts, it might be worthwhile to explore this further and strengthen your hypothesis in an other unique kidney cohort.

**Limitations:**
1. The abstract would benefit from some clearer emphasis on the clinical implications of the findings.
2. For reproducibility, the criteria for kidney sample selection, especially distinguishing between CKD and non-CKD donors, should be explicitly detailed.
3. While the differential susceptibility of kidney cell types to LOY is intriguing, the mechanisms underlying these observations warrant further exploration.
4. The established correlation between LOY and CKD progression is noteworthy. However, translating this into clinical implications remains ambiguous. It would be beneficial to elucidate how this information can be integrated into patient care or prognosis, even if the target audience of this journal is primarily basic researchers.
5. A comparative analysis with other studies investigating LOY in different tissues or organs would enhance the paper's depth. Such a comparison would determine whether the observed patterns are kidney-centric or more generalized.
6. The proposition that LOY might serve as a marker of cellular senescence is compelling. Yet, the study could delve deeper into this aspect, potentially exploring interventions to counteract the effects of LOY on CKD progression.

**Authors’ response**

Reviewer #1: In the manuscript entitled, "Mosaic loss of Y chromosome is associated with aging and epithelial injury in chronic kidney disease" Wilson et al. demonstrate a connection between Y chromosome loss, kidney cell maladaptive repair, and CKD. Although the manuscript is largely technical in nature, demonstrating various transcriptomics techniques can measure LOY, the findings are of interest and have the potential to have a significant impact on the field. The study is well designed, and the data are convincing. That said, for these techniques to show their true value, they need to be compared to the gold standard. Karyotype analysis to ensure the core findings, deletion of the Y chromosome, are reproducible would greatly strengthen the study.

These and other concerns are listed below:

1. In the introduction the Authors state, "we describe an approach to detect LOY and other mCA by single cell sequencing." Although the findings are interesting and of potential significance, the scRNA seq technique is not without limitations. If this is to be a novel technique, it should be compared to the gold standard of monitoring LOY, karyotyping, to validate the findings. This doesn't have to be a large number of samples, just enough to confirm LOY is significantly more common in CKD.

We agree that an additional method would be useful to validate single-cell LOY estimates. Digital PCR has been used by other investigators to detect LOY in peripheral blood (Danielsson et al. 2020 Eur J Hum Genet, Jiang et al 2023 BMC Psychiatry) and we developed a custom multiplex digital PCR assay for accurate detection of LOY in the kidney. We used Digital PCR because it is compatible with snap-frozen tissue and does not require actively dividing cells like a standard karyotype. We used the Absolute Q Quantstudio System which uses microfluidics to disperse a digital PCR reaction into 20,000 individual reactions in separate microwells. Each of these microwells amplifies a Taqman probe targeting a region on the Y chromosome or a reference probe on the X chromosome – each with a different fluorophore. We can estimate LOY by calculating the ratio of microwells that amplify the Y chromosome target sequence relative to the X chromosome reference. We validated the assay by performing a serial dilution of male:female DNA using cell culture lines to simulate LOY (see figure 4A). In this manner, increasing the proportion of female cells decreases the concentration of the Y chromosome target sequence relative to the X chromosome reference sequence. For example, a 50:50 mixture of male:female DNA would result in a ratio of 1:3 for Y:X chromosomes and an expected copy ratio of 0.33. Our assay is accurate and reproducible with a coefficient of variation on the order of a few percent (see figure 4A). Next, we isolated gDNA from kidney cortex of five male samples that were also analyzed by single-cell sequencing. These samples included two male multiomes and three snATAC-seq libraries. When we performed dPCR on these samples, all five showed a reduction in the ratio of chrY:chrX (see figure 4B). These data suggest that dPCR can detect LOY and that the dPCR and single-cell estimates are in the same range for tissues taken from the same donor. Furthermore, we compared dPCR LOY estimates with the single-cell estimates and found a significant positive correlation (see figure 4C). We have prepared these data as a new figure (#4) in the main manuscript. We recognize that this does not prove that LOY is significantly more common in CKD, however, we would need a larger sample size for that analysis because we have to account for confounding factors like age. We have added additional discussion to our manuscript to emphasize this point.

2. Is the Y chromosome truly 'lost' or just silenced? For instance, in Figure 3D it is shown LOY is associated with cell division. Is it known whether the Y chromosome is silenced in cell division? This could be addressed in the data from #1 above.

We developed a dPCR assay to measure LOY and found a significant positive correlation with our single-cell estimates (see response to reviewer comment 1 above). These data support the idea that the Y chromosome is truly lost and not just silenced. We recognize the limitations in analyzing sparse single-cell datasets for LOY and have added discussion to emphasize this point.

3. There is very little discussion of the limitations of this study. At least some of the data was based on a small number of patients, i.e. n=4 or 5, which could be a limitation. Also, as the paper demonstrates the utility of transcriptomics in analyzing LOY, limitations to the various transcriptomics approaches used should be discussed.

We recognize that the multiome analysis had relatively few male samples and have added additional discussion surrounding the limitations of this sample size. However, we did support the multiome analysis with additional analyses in larger snATAC-seq and sc/snRNA-seq datasets. We have added discussion to describe the limitations of sc/snRNA-seq analysis and detection of LOY. In short, it is critical that the sequencing depth of the libraries is sufficient to control the false positive rate.

4. It would be interesting to compare the gene ontology pathway enrichment data in 1F and DEG analysis in 3D to cells from female patients, as these cells never had an Y chromosome.

We compared male LOY to female cells to generate a list of differentially expressed genes (DEGs) for each cell type in the multiome dataset. We used this list of DEGs to do a gene set enrichment analysis for the proximal convoluted tubule (PCT). The enriched pathways include a variety of ion transport and metabolic pathways, but did not include apoptosis or chromatin assembly pathways observed in the LOY vs. XY comparison. We hypothesize that the DEGs that differentiate LOY from female cells are predominantly due to sexual dimorphism in the proximal tubule, which has been recently described by single-cell sequencing (Xiong et al. Developmental Cell 2023). We also compared male LOY to female cells in the KPMP sc/snRNA-seq dataset to see if DNA repair or G1/S Transition genes are differentially expressed. Similar to the LOY vs. XY comparison in Figure 3D, LOY cells showed increased expression of BCL2 and EGFR when compared to female cells. Overall, the transcriptional changes are highly similar and suggest that LOY cells show similar changes in DNA repair pathways when compared to either male or female cells.

5. Why is LOY worse than never having an Y chromosome in the first place? The data in 3A and SF7 seem to indicate both males and females have PT_VCAM1, PT_PROM1 and PT_MT cell populations at relatively similar rates, but in males, these cells are more likely to have LOY. The Authors should discuss what it is about the LOY that is different than having XX chromosomes.

We appreciate this comment and have added additional discussion to the manuscript to outline the differences between male LOY and female XX cells. Indeed, both male and female kidneys have PT_VCAM1, PT_PROM1 and PT_MT cells and it is unclear what factors contribute to the emergence of these populations. We hypothesize that these cells have increased DNA damage relative to healthy proximal tubule and one of the manifestations of increased DNA damage is LOY. We have not found a similar somatic mutation in female cells, but it is possible that there are other mutations that we cannot detect. For example, mosaic loss of the X chromosome (LOX) increases in aging women in blood. However, LOX predominantly affects the inactive X chromosome, which is neither transcribed nor accessible by our methods. Future studies may benefit from library modifications or improved algorithms that can detect LOX and other difficult-to-detect somatic mutations (eg. copy-neutral loss-of- heterozygosity, somatic SNV, mitochondrial mutations) that may be more prevalent than anticipated.

The Y chromosome has important functions independent of sex determination that distinguish them from XX cells. For example, LOY helps bladder epithelial cells evade the immune system (Abdel-Hafiz et al. Nature 2023) and blood cells evolve into leukemia (Zhange et al. JCI Insight 2022). It is unclear if female cells can carry out the same functions without a Y chromosome, but it may be related to the gene dosage effect of having two X chromosomes or alternative pathways on autosomes.

6. The data in Figure 4 doesn't seem to add much to the current study. There is no mention if the samples are from males or females and there is no connection to the Y chromosome. If staining for the Y chromosome was done prior to the RNA analysis, it could have perhaps strengthened the data.

We recognize that the spatial data is not single-cell resolution, however, we do think that it helps to identify microenvironments that are enriched for the same cell markers expressed in injured proximal tubule populations. We have added additional information for the sex of the spatial libraries to our manuscript.

We isolated gDNA from four of the male spatial libraries and performed dPCR to evaluate LOY. Two of the libraries had sufficient DNA for our assay and both of these libraries measured a decreased ratio of chrY:chrX – indicating that there was LOY within the samples. We did not include this data in the manuscript because two of four samples had insufficient DNA for our assay. However, we do believe that this is an exciting area for future research.

7. Although there is a clear connection between LOY and kidney failed repair phenotype, it is not clear which comes first. The fact that the majority of the PT_VCAM1 group retains the Y chromosome suggests the loss is occurring after the cells reach the maladaptive repair stage. Also, PT_VCAM1 cells are only approximately twice as likely to have LOY compared to PT group. That said, it is unclear why this small population of maladaptively repaired cells contributes to kidney functional decline in the first place. It is possible that the LOY worsens the failed repair phenotype in this subpopulation. It would be interesting further analyze the DEG to determine if there is a change in failed repair vs failed repair + LOY. In other words, a PT_VCAM1-LOY vs PT_VCAM1-XY (or PT_PROM1-LOY vs PT_PROM1-XY) analysis.

We agree that it is difficult to determine whether LOY happens before or after the cells adopt a failed repair phenotype. Nevertheless, LOY does have a unique transcriptional signature among failed repair cells. We compared PT_VCAM1-LOY vs PT_VCAM1-XY and PT_PROM1-LOY vs PT_PROM1-XY and observed similar enriched pathways (see supplemental figure 9), including altered DNA binding, acid transport, tight junction assembly, and metabolic functions. As the reviewer pointed out, these data suggest that LOY may worsen the failed repair phenotype. We have added these data as new supplemental figure (#9) to our manuscript.

Minor:

1. The "Proportion LOY" data, 1E, 2C, 2C, and etc, isn't clear. Does each dot in a given condition represent a different patient? If so, the numbers don't match the stated n values.

Thank you for this suggestion. Some of the points were overlapping and not visible in the figure so we have separated the points. In addition, we initially excluded a minority of points that had fewer than 50 cells. We felt that the estimated proportion of LOY may be inaccurate when the cell population is not adequately sampled. The new figures now show all of the point estimates for male cell types and samples regardless of the number of cells per sample. The old and updated figures are largely similar and we have now included all of the point estimates in our new figures.

2. It is somewhat confusing to understand which data sets contain data from men and women and which ones are men only. For instance, in the results description for Figure 2 it is stated, "we analyzed 167,772 nuclei from 22 donors, including both men (n=12) and women (n=10)." But a few lines later, "We evaluated male cells for LOY." I take from this only 2A has male and female data, but it is not completely clear. Specifying male vs female or including the n values in the figure legends could make this easier to follow.

We have updated all of the figure legends to be clear which figures are analyzing male and female data. That is correct that figure 2A is the only panel that has both male and female data because the remaining panels are focused on LOY.

Reviewer #2: This study examines loss of the Y chromosome (LOY) on single cell RNA and ATAC sequencing in the kidney. The authors speculate that LOY is associated with many phenotypes/outcomes, but no direct measurements are made. For example, claims are made that LOY is an indicator of DNA damage, senescence and disease status, but independent markers for DNA damage and senescence are not measured and a comparison between normal and disease kidney is not made. Specific comments are below:

The association between age and LOY is widely recognized. The current analysis with the kidney dataset was performed at the end of this manuscript and apparently not incorporated into previous analyses of differential gene expression. Thus, it appears that the data does not adjust for age when the association of LOY with changes in gene expression are made. Hence, age can be a confounding factor in the outcomes that are attributed to LOY. In other words: Are the identified pathways caused by LOY? Or are they pathways that increase with age, which is then over-represented in the LOY populations?

This is an excellent point and we have revisited our differential expression analyses to adjust for age. In the multiomes, adjustment for age reduced the number of differentially expressed genes across all cell types from 1,030 to 477 when comparing LOY to XY. The proximal convoluted tubule (PCT) was a notable exception and had an increase in the number of DEGs from 218 to 321 after age adjustment. We used the updated list of 321 significant DEGs from PCT and performed GSEA as previously- described (see figure 1F updated). Many of the same pathways from the unadjusted analysis were retained in this new analysis, including apoptotic processes, chromatin organization, and mitotic division among others. We have incorporated this updated figure and tables into our manuscript.

We also adjusted the KPMP differential expression analysis for age using the same approach. In the KPMP dataset, adjustment for age reduced the number of differentially expressed genes across all cell types from 23,875 to 18,399. We used the updated list of DEGs to reexamine DNA double strand break repair and G1/S transition pathways (see updated 3D). All 23 of the DSB repair genes retained statistical significance after adjusting for age. A total of 27 of 32 significant DEGs from the unadjusted analysis were also significant in the age-adjusted G1/S transition analysis. One notable absence was EGFR, which was not statistically significant in proximal tubule after adjusting for age in the KPMP dataset. We have now incorporated this new figure into our manuscript.

The connection between LOY and proliferation in various cell types is based on anecdotal evidence of proliferation. Proliferation is not directly (or indirectly) assessed. For example, were proliferation/cell cycle genes differentially expressed in LOY- positive vs. LOY-negative cells within a particular cell type? This would at least provide indirect support for this claim - that is currently unsubstantiated.

This is a very important question that our single-cell dataset may not be able to answer. We evaluated hallmark cell cycle genes for differential expression between LOY and XY cells across all cell types (see supplemental figure 10). In general, LOY was associated with reduced expression of cell cycle genes (blue color), suggesting that they are not actively dividing. These data support the notion that LOY cells are senescent, but they do not give any information about whether different cell types have divided more or less during their lifetime. In order to answer that question, we would need a more direct marker of cell division like lineage tracing. In our manuscript, we hypothesized that PT_VCAM1, PT_PROM1 and other injured proximal tubule cell types may have undergone more cell divisions than non-injured PT. We do not have direct evidence that this is the case and have made that more clear in our discussion.

However, we do think an increase in the number of cell divisions is a likely explanation for the increased probability of a cell losing the Y chromosome.

Results presented here suggest that LOY in proximal tubule cells is associated with large transcriptional changes related to cell damage, but the overall conclusion put forth is that LOY is a marker for damaged cells. Could LOY be directly causing these transcriptional changes? Within the PT_VCAM1 population (indicative of damaged cells), what are the differentially expressed genes and transcription factor activities? At a single cell level can you compare DNA damage-associated gene expression in LOY- negative vs. LOY-positive cells?

We are very interested in this question and believe that LOY may be both a marker of DNA damage and also an effector of transcriptional changes. There is recent literature that has studied LOY in bladder cancer (Abdel-Hafiz et al. Nature 2023). They report that LOY cells have altered interactions with the adaptive immune system and increased proliferative rate. We hypothesize that LOY may have independent effects in the kidney too. When comparing PT_VCAM1-LOY to PT_VCAM1-XY or PT_PROM1- LOY to PT_PROM1-XY, we identify differentially expressed genes and transcription factor activities. In the KPMP dataset, there are 240 DEGs that meet the age-adjusted pval threshold and 1754 that meet the unadjusted pval threshold. We did a GSEA of the DEGs for the KPMP dataset (see supplemental figure 9) and identified some similarities in developmental pathways and cell motility pathways.

We also compared the transcription factor activities for PT_VCAM1-LOY vs PT_VCAM1-XY for the multiomes and snATAC-seq datasets. There was a short list of transcription factors that had differential activity for both the multiomes and ATAC datasets. This list included HNF1A, HNF1B and NFIC and each of these TF showed decreased activity in PT_VCAM1 LOY vs XY. In particular, we hypothesize that HNF1A and HNF1B are two TF that may drive transcriptional changes between LOY vs XY cells.

At a single cell level can you compare DNA damage-associated gene expression in LOY-negative vs. LOY-positive cells?

We evaluated a list of DNA damage-associated genes for differential expression in the multiome and KPMP datasets. The multiomes returned no DNA damage-associated DEGs, which may be due to reduced power in this dataset. In the KPMP dataset, we identified a number of DNA damage-associated DEGs (see supplemental figure 9) associated with LOY in multiple cell types. The majority of genes were dowregulated (blue color) and one of the genes present in multiple cell types was REV3L. REV3L encodes the zeta catalytic subunit of DNA polymerase and inhibition of REV3L has been associated with persistent DNA damage and growth arrest in several cancer cell lines (Yang et al. PLoS One 2015, PMID: 25781640).

Studies here are focused on proximal tubule cells, but many other cell types are present in the kidney and could have connections to LOY. In particular, leukocytes have a well-documented LOY burden, and epidemiological studies have provided associations between LOY in blood and many diseases. Except with cancer, these epidemiological associations of LOY with disease do not exist with respect to non- leukocyte cell types, emphasizing the importance of kidney leukocyte analyses.

We agree that there are other cell types in the kidney that are also affected by LOY. We analyzed the proximal tubule because it is abundant in the kidney cortex (and single cell datasets) and it has a high proportion of LOY and recognize that this is a form of selection bias. However, other segments of the nephron have also been shown to have an injured subtype in single cell datasets, particularly the thick ascending limb of the loop of Henle. The thick ascending limb (TAL1, TAL2) also had an increased proportion of LOY compared to other cell types. TAL1 and TAL2 are adjacent segments in the thick ascending limb, and when we compared LOY vs XY cells in either segment we identified a number of DEGs in both the multiomes and KPMP analysis (Supplemental Data 2, 3, 8, 9).

The number of leukocytes in our dataset is limited, but we were able to reanalyze a publicly-available single-cell dataset of leukocytes from the kidney. This dataset includes matched CD45-enriched samples of peripheral blood, non-tumor kidney, and renal cell carcinoma from seven male and one female donor (Kourtis et al. Nat Cancer 2022). We analyzed the snATAC-seq libraries from seven male donors to determine the relative proportion of LOY among leukocytes in the kidney and peripheral blood. Overall, leukocytes had a small proportion of LOY in peripheral blood, kidney and tumor-associated leukocytes (see supplemental figure 7). The estimated LOY proportion ranged from 0-2% depending on the cell type and there was somewhat greater LOY in B and T cells (see panel D). We compared the CD45-enriched LOY estimates to our snATAC-seq data, which had a somewhat higher LOY estimate on the order of 5-6%. This may be due to the lower age of the donors from this study (57 years) or the variable cell composition compared to our snATAC-seq datasets. It may be beneficial in future studies to investigate kidney leukocyte populations from older individuals to evaluate the connection between LOY in leukocytes and kidney disease progression. We have incorporated this new figure into our supplemental data (#7).

The spatial transcriptomics analysis does not have the resolution to determine LOY cells (as pointed out in the results).

We appreciate this comment and recognize our spatial analysis lacks the resolution to detect LOY. However, we do feel that it helps to identify pro-fibrotic microenvironments that express many of the same markers we see in injured proximal tubule cells. We performed dPCR to evaluate LOY in the male spatial libraries and in two of the samples we confirmed LOY. This will be an exciting area for future work.

Sample sizes with cells and patients include men and women, but of course cells from women were not analyzed in downstream analyses on LOY. Thus, it seems that reported sample sizes are misleading.

We appreciate this comment and have updated all of the figure legends and text to be clear about the sample size for individual analyses.

Transcription factor activity based on open chromatin and transcription factor expression based on RNA profile appear to be confused and are used interchangeably throughout the results.

We appreciate the close attention to detail and have updated all of these references to refer to transcription factor activity.

These authors use their own generated dataset and supplement with other publicly available datasets, but no attempt was made to harmonize the data and combine datasets (that would promote robustness of some conclusions).

We thank the reviewer for this important point and have now integrated the multiome and kpmp datasets to do a joint differential expression analysis. We have included additional discussion on this integrated analysis in the section titled “Age and mCA are associated with LOY in the kidney”. We used label transfer to assign cell type identities to the kpmp dataset and harmonize annotations. The integrated object included 185,723 cells of which 113,445 were male. We compared LOY vs XY for each cell type to identify differentially expressed genes. In total, there were 2,270 differentially expressed genes across all cell types that met the bonferroni adjusted pval threshold < 0.05. We have now included this table in our supplemental data and added additional description of the results to the manuscript. Some highlights include increased power to detect differentially expressed genes in the leukocyte populations. Within the PT- VCAM1-LOY vs PT-VCAM1-XY comparison, we identified 90 differentially expressed genes that met the adjusted threshold. These genes were enriched in apoptosis, adhesion, and developmental signaling pathways.

We also conducted an age-adjusted differential expression analysis in the integrated dataset. After age-adjustment, there were 2,235 differentially expressed genes across all cell types. 1,929 of these genes were overlapping in the unadjusted and age- adjusted differential analyses, which points towards robustness of the analysis.

We would also like to point out that the figure entitled “Age and mCA as Predictors for LOY” is a harmonized analysis of the sc/snRNA-seq, snATAC-seq and multiome datasets. Panel A examines the role of age and LOY and represents a combination all three datasets. Panel B-E examines the role of mCA and is a joint analysis of all male snATAC-seq data.

Reviewer #3:

I reviewed the article by Wilson et al. The authors delve into several pertinent questions:

1. The association of LOY with kidney disease.

2. The relationship of LOY to the aging kidney.

3. The cell type where LOY is most prevalent.

4. The chromatin accessibility profile in PT cells with LOY.

5. The transcriptome of PT cells with LOY.

6. The transcriptome of spatial neighborhoods in and around the PT.

While the paper adopts a primarily descriptive approach, this is by no means a limitation. Descriptive studies are foundational in science, often paving the way for hypothesis-driven research. The authors convincingly demonstrate an association between LOY and the aging kidney. It's particularly noteworthy that proximal tubular cells from older kidneys exhibit a higher proportion of LOY. Additionally, adjusted for age, the proximal tubule was identified as the cell type with the highest percentage of cells with LOY.

While not directly studied here, it would be insightful to understand if LOY has been studied in the context of kidney transplantation. Factors such as immune processes, immune reperfusion injury, drug toxicity, and DGF (particularly ATN) might influence the prevalence of LOY ?. This is only pertinent for patients receiving kidneys from male donors. Given that your group possesses data on transplant cohorts, it might be worthwhile to explore this further and strengthen your hypothesis in an other unique kidney cohort.

We appreciate the reviewer comment and have interrogated publicly-available datasets to evaluate kidney allografts for LOY. We have a limited number of male donor allografts and were only able to evaluate two samples. The male donor allografts had evidence of LOY in a somewhat larger proportion than the native kidneys. We did observe an increased proportion of PT_VCAM1 LOY cells compared to the proximal tubule, however, many of the cell types had elevated proportions of LOY. It is unclear if this is related to allograft injury, sparse sampling inherent to scRNA-seq or limited sample size. We feel that this is an exciting new direction for research and defer to future studies.

**Limitations:**

1. The abstract would benefit from some clearer emphasis on the clinical implications of the findings.

We have added additional discussion surrounding clinical implications to the abstract. Potential clinical implications might include prognostic value for quantifying LOY in kidney (or other tissues). Our digital PCR assay may be useful in that regard. In general, mCA and somatic mutational burden may be important predictors of disease progression, cancer risk, or response to therapy.

2. For reproducibility, the criteria for kidney sample selection, especially distinguishing between CKD and non-CKD donors, should be explicitly detailed.

We have added additional details for how CKD and non-CKD donors were distinguished to our methods section. In brief, we selected donors with an eGFR < 60 according to KDIGO guidelines.

3. While the differential susceptibility of kidney cell types to LOY is intriguing, the mechanisms underlying these observations warrant further exploration.

We appreciate this comment and agree that future studies would benefit from further exploring the mechanism for LOY. To date, the exact mechanism for LOY (in any tissue) is unknown. It may be related to missegregation of chromosomes during cell division. This is supported by the observation that many of the genes that confer heritability of LOY (and LOX) are involved in cell cycle and mitosis. There are a variety of models that have been proposed, including micronucleation, telomere attrition, and centromere dysfunction.

4. The established correlation between LOY and CKD progression is noteworthy. However, translating this into clinical implications remains ambiguous. It would be beneficial to elucidate how this information can be integrated into patient care or prognosis, even if the target audience of this journal is primarily basic researchers.

We appreciate this reviewer comment and view LOY as a potential biomarker of DNA damage. It’s possible that LOY or mCA burden will have prognostic value that determines the rate of CKD progression or future cancer risk. If cells with a high mCA burden are truly pro-fibrotic then it may be beneficial to design therapies to eliminate them. We have added discussion to the manuscript to further elaborate on potential clinical applications for LOY or mCA detection.

5. A comparative analysis with other studies investigating LOY in different tissues or organs would enhance the paper's depth. Such a comparison would determine whether the observed patterns are kidney-centric or more generalized.

We agree that it will be helpful to understand how LOY affects different tissues throughout the body. The only paper we are aware of that has examined LOY in multiple tissues is an aging study in rats. This study showed that the brain, liver, kidney, blood and testis were susceptible to LOY whereas other tissues were not. In humans, LOY analysis has been mostly focused on blood and bone marrow – although there have been recent publications in brain and bladder. We have added additional discussion to the manuscript to outline studies in humans and animals to provide better context for tissue-specific LOY. The major takeaway points from these studies is that the rate of LOY is tissue- dependent and LOY cells have a unique transcriptional signature in multiple tissues and cell types

.

6. The proposition that LOY might serve as a marker of cellular senescence is compelling. Yet, the study could delve deeper into this aspect, potentially exploring interventions to counteract the effects of LOY on CKD progression.

We agree that it may be beneficial to design therapies that target senescent cells in the kidney. In particular, targeting pro-survival pathways in LOY cells may promote apoptosis and help to eliminate them. There is limited data on the utility of targeting these pathways, but it will be important to avoid off-target effects. For example, inhibition of BCL2 may adversely affect kidney homeostasis (Borkan et al. Sem Neph 2016). It may be necessary to develop selective inhibitors that target pathways unique to senescent cells. An alternative approach would be to counteract the pro- inflammatory effects of senescent cells by inhibiting inflammatory pathways. There is a wide literature describing the benefits of immune system inhibition in mitigating the response to acute kidney injury or CKD progression, however, these studies did not necessarily examine whether senescent cells are a primary source of inflammatory signals. We have added additional discussion to our manuscript to describe potential interventions.

**2^nd^ round**

**Reviewer 1**

The Authors have satisfactorily addressed all of my concerns.

**Reviewer 3**

thank you for your clarifications and additions. I look forward to further work from the group
